# Computing energy landscape maps and structural excursions of proteins

**DOI:** 10.1186/s12864-016-2798-8

**Published:** 2016-08-18

**Authors:** Emmanuel Sapin, Daniel B. Carr, Kenneth A. De Jong, Amarda Shehu

**Affiliations:** 1 Department of Computer Science, George Mason University, 4400 University Drive, Fairfax, 22030 VA USA; 2 Department of Statistics, George Mason University, 4400 University Drive, Fairfax, 22030 VA USA; 3 Krasnow Institute for Advanced Study, George Mason University, 4400 University Drive, Fairfax, 22030 VA USA; 4 Department of Bioengineering, George Mason University, 4400 University Drive, Fairfax, 22030 VA USA; 5 School of Systems Biology, George Mason University, 10900 University Boulevard, Manassas, 20110 VA USA

**Keywords:** Protein equilibrium dynamics, Multi-state protein, Multi-basin energy landscape, Energy landscape map, Sample-based representation, Evolutionary algorithm, Structural excursion, Mechanical work, Nearest-neighbor graph, Low-cost paths

## Abstract

**Background:**

Structural excursions of a protein at equilibrium are key to biomolecular recognition and function modulation. Protein modeling research is driven by the need to aid wet laboratories in characterizing equilibrium protein dynamics. In principle, structural excursions of a protein can be directly observed via simulation of its dynamics, but the disparate temporal scales involved in such excursions make this approach computationally impractical. On the other hand, an informative representation of the structure space available to a protein at equilibrium can be obtained efficiently via stochastic optimization, but this approach does not directly yield information on equilibrium dynamics.

**Methods:**

We present here a novel methodology that first builds a multi-dimensional map of the energy landscape that underlies the structure space of a given protein and then queries the computed map for energetically-feasible excursions between structures of interest. An evolutionary algorithm builds such maps with a practical computational budget. Graphical techniques analyze a computed multi-dimensional map and expose interesting features of an energy landscape, such as basins and barriers. A path searching algorithm then queries a nearest-neighbor graph representation of a computed map for energetically-feasible basin-to-basin excursions.

**Results:**

Evaluation is conducted on intrinsically-dynamic proteins of importance in human biology and disease. Visual statistical analysis of the maps of energy landscapes computed by the proposed methodology reveals features already captured in the wet laboratory, as well as new features indicative of interesting, unknown thermodynamically-stable and semi-stable regions of the equilibrium structure space. Comparison of maps and structural excursions computed by the proposed methodology on sequence variants of a protein sheds light on the role of equilibrium structure and dynamics in the sequence-function relationship.

**Conclusions:**

Applications show that the proposed methodology is effective at locating basins in complex energy landscapes and computing basin-basin excursions of a protein with a practical computational budget. While the actual temporal scales spanned by a structural excursion cannot be directly obtained due to the foregoing of simulation of dynamics, hypotheses can be formulated regarding the impact of sequence mutations on protein function. These hypotheses are valuable in instigating further research in wet laboratories.

**Electronic supplementary material:**

The online version of this article (doi:10.1186/s12864-016-2798-8) contains supplementary material, which is available to authorized users.

## Background

Experimental, theoretical, and computational studies have shown that protein function is the result of a complex yet precise relationship between protein structure and dynamics [1–3]. Long gone are the days when proteins were viewed as rigid molecules [4], with the atom nuclei frozen in specific positions in the three-dimensional (3D) structural models captured by X-ray crystallography [5]. Nowadays, wet-laboratory techniques based on single-molecule fluorescence spectroscopy provide irrefutable evidence of proteins as macromolecules in perpetual motion [2], even catching proteins in the act of switching between different structures to bind different molecular partners [6]. The ability of a protein to switch between different structures under physiological conditions (at equilibrium) is key to biomolecular recognition and function modulation [7]. This finding warrants characterizing the equilibrium structural dynamics of a protein as a means of exposing the range of activities of a protein in the cell [8].

While significant advances have been made in the wet laboratory [6, 9–12], existing techniques are in principle limited by the disparate spatio-temporal scales involved in protein dynamics; proteins undergo small (sub-angstrom) and large (>10Å) structural changes at different temporal scales, spanning from a few femto-seconds to milli-seconds and more [13]. Dwell times of proteins in specific structural states may be too short to be detected in the wet laboratory.

Computational methods that simulate the constrained dynamics of the bonded atomic particles in a protein molecule via iterative application of Newton’s laws of motions are appealing. By following motions of atoms along the negative gradient of a molecular mechanics force field, these methods, also known as Molecular Dynamics (MD) methods, directly simulate structural excursions of a protein [14]. Since energy landscapes are highly multi-dimensional [15] (directly related to both independent and concerted motions of thousands or more atoms comprising a protein molecule), MD methods have to be operated in a random-restart fashion to sufficiently explore the structure space accessed by a protein at equilibrium. Typical computational efforts can exceed several weeks on large (several-hundred core) supercomputers [16] for medium-size proteins (100−300 amino-acids long), though advances in hardware and specialized architectures are beginning to broaden the scope and scale of MD methods to larger macromolecular assemblies and even viral capsids [17, 18].

The challenges regarding characterization of equilibrium protein dynamics are better understood from a protein energy landscape perspective, which links protein structure, dynamics, and function [19]. Briefly, measuring the extent to which a structure satisfies the (physical) constraints that atoms place on one another allows one to associate an energy landscape with the structure space of a protein. Structural excursions of a protein at equilibrium correspond to hops between energy basins in the landscape [20]. A basin, visually corresponding to a valley in the energy landscape, contains structures with similar energies. The set of structures mapped to the same basin represent a particular protein state. These states can be thermodynamically-stable or semi-stable, depending on the width and depth of the corresponding basin. A protein may spend more time in a wider and deeper basin (the state is stable) than in a narrower and shallower basin (the state is semi-stable) [20]. Energy barriers between basins regulate the time it takes for a protein to switch between basins [7, 20]. The interested reader is referred to works in [1, 21] for detailed reviews of energy landscapes and motions of proteins.

The energy landscape view clarifies why a complete and detailed account of protein equilibrium dynamics is a non-trivial task. In principle, the task requires a comprehensive characterization of both the protein structure space and the underlying energy landscape that governs the accessibility of structures and excursions between them at equilibrium. While wet-laboratory studies may not catch semi-stable structural states (due to insufficiently long dwell times), computational approaches that simulate protein dynamics quickly become computationally intractable for even moderate-size proteins.

In this paper we present a novel computational methodology that takes a complementary approach to the MD-based approach. While the goal remains to elucidate the equilibrium dynamics of a protein by computing structural excursions at equilibrium, the dynamics are not simulated directly. Instead, a two-step approach is followed. *First*, stochastic optimization (randomized/stochastic search) is employed to explore a protein’s structure space and construct a map of the energy landscape relevant for equilibrium dynamics. *Second*, the map is analyzed and queried for paths of intermediate structures that link two structural states of interest, effectively yielding an ensemble of paths that provide an on-demand view of the equilibrium dynamics relevant to a specific structural excursion. This two-step approach foregoes any direct information on the temporal scales involved, as there is no notion of physical time in the computed structures and paths connecting them. However, by doing so, the computational demands become much more reasonable; for instance, investigations of medium-size proteins of 150 or more amino acids can be conducted on small clusters (of no more than 16 CPUs) in no more than a few days (ranging from 7 to 15). Moreover, during this process, close to a million structures are generated, embedded in multi-dimensional maps of energy landscapes, and available to answer queries about energetically-feasible structural excursions at equilibrium between any two structures of interest.

The advantages of stochastic over systematic search to explore high-dimensional variable spaces have been demonstrated in various domains. In protein structure modeling, algorithms that navigate the structure space of a protein via the Monte Carlo (MC) approach have been shown to have higher exploration capability than MD-based ones [22]. Furthermore, evolutionary algorithms (EAs) have been shown to provide significant improvements over MC-based algorithms [23, 24]. Specifically, for *d*e novo protein structure prediction, EAs with domain-specific insight have been shown to rapidly locate the global minimum and reproduce the native structure [25, 26]. However, when the focus is on multi-state proteins with complex multi-basin energy landscapes, the objective goes beyond rapidly locating one structural state and requires an exploration of the breadth of the structure space. Recent evolutionary search techniques have advanced efforts in this direction [27–29].

A key starting point of recent work is the increasingly rich set of structural data for both the wildtype (WT) and variants of multi-state proteins being deposited by wet-laboratory scientists in the Protein Data Bank (PDB) [30]. Work in [31] has shown that a statistical characterization of this structural information provides important and useful information about the structure space of a particular protein. A simple, generational EA operationalizes this idea in [27]. Work in [28] enhances the exploration capability of the EA while still operating under the classic optimization setting of finding the global minimum (via a decentralized selection operator that delays population take-over by the most fit individuals). Work in [29] finally switches from the classic setting to that of obtaining a comprehensive map of a (multi-state) protein’s multi-basin energy landscape via the concept of the hall of fame. The resulting EA is shown able to map complex, multi-basin fitness landscapes beyond the protein modeling domain via careful combination of local and global search [32].

These collective algorithmic developments have now made it possible to build comprehensive and detailed maps of energy landscapes of medium-size proteins with a modest computational budget. The EA we employ for mapping protein energy landscapes in the methodology proposed here builds on all these previously-published evolutionary search techniques to effectively and efficiently map the structure space available to a protein at equilibrium. The “Methods” Section summarizes this EA for the sake of completeness, paying particular attention to those aspects that give it the ability to efficiently map energy landscapes of medium-size proteins.

Analysis of maps computed to represent energy landscapes is non-trivial. Even when the focus is simply to locate basins, the analysis involves several hundred thousands of multi-dimensional data points that reside in a highly non-linear landscape. Past work [28, 29, 33] has relied on visual analysis of 2D projections of all structures ever computed during the execution of an EA or only those structures in the hall of fame/map. We make the case in the “Results” Section that such analysis is informative, but the projection can sacrifice possibly interesting energetic features in the multi-dimensional map. Hence, in this paper we utilize additional graphical techniques to visualize and analyze the computed multi-dimensional maps. The techniques reveal not only basins already captured in the wet laboratory, but also new energetic features indicative of interesting, unknown thermodynamically-stable and semi-stable regions of the equilibrium structure space.

Mapping the energy landscape of a protein provides an opportunity to extract information on its equilibrium dynamics in much in the same way the map of a city allows extracting information on routes connecting landmarks. In previous work [28, 29, 33, 34], we have relied on qualitative summarizations of protein dynamics based on the location of energy barriers and other features of a mapped landscape, and how these features differ in the variant forms of a protein. Here we propose a procedure to extract information on the equilibrium dynamics of a protein by computing structural excursions between structures of interest. The procedure builds on ideas utilized in robotics-inspired methods for protein motion computation [35–37]. In these methods, structures are embedded in a nearest-neighbor graph (referred to as a roadmap), which is then queried for a path connecting a start to a goal structure structure. In this paper, the structures are those produced by an EA mapping process. That is, they constitute a comprehensive and detailed map of the energy landscape. Care has to be taken to embed them in a nearest-neighbor graph and utilize them for path queries. Moreover, unlike related work in robotics-inspired modeling, where the focus is typically on one path, the procedure proposed here reveals an ensemble of energetically-similar paths. This focus is warranted in order to obtain a broader view of the stochastic but energy-driven nature of protein structural excursions (and equilibrium dynamics).

The methodology proposed in this paper to build maps, analyze them, and then query them for structural excursions is applied to several proteins of importance in human biology and disease. In addition, detailed comparison of the maps and path ensembles is conducted on the WT and 7 variant sequences of an oncogenic protein. This comparative setting evaluates the ability of the proposed methodology to explain the impact of mutations on protein equilibrium dynamics and in turn on misfunction. These results are presented in the “Results” Section, and a discussion of how they reproduce, explain, or further existing knowledge is provided in the “Discussion” Section.

While the actual temporal scales spanned by a modeled structural excursion cannot be obtained by the proposed methodology due to the foregoing of simulation of dynamics, specific hypotheses can be formulated nonetheless regarding the impact of sequence mutations on function. These hypotheses are valuable in instigating further research on structure-function studies in wet laboratories. The advantages and disadvantages of the proposed methodology, as well as possible directions of further research, are summarized in the “Conclusions” Section, which concludes this paper.

## Methods

The input to the proposed methodology consists of a protein sequence *α*, a set of structures $\mathcal {S}_{\text {PDB}}$ representing stable structural states for sequences no more than 3 amino acids different from *α*, and (a pair of start and goal) structures of interest for a possible excursion. The methodology first performs a principal component analysis (PCA) on the structures in $\mathcal {S}_{\text {PDB}}$ in order to define a low-dimensional representation of the protein structure space. An evolutionary algorithm (EA) is then applied to this PCA-defined space to construct a map representing the all-atom energy landscape of *α*. Finally, a path-finding algorithm uses this map to compute energetically-feasible paths realizing structural excursions of interest. The methodology is shown in pseudocode in Algorithm 1.

Below we first relate details on the principle that allows utilizing structures in $\mathcal {S}_{\text {PDB}}$ to define the (reduced) variable space underlying the structure space of a protein sequence of interest *α*, as well as describes the technique employed to do so (lines 1–2 in Algorithm 1). The EA that explores this variable space to build a multi-dimensional map of the all-atom energy landscape of *α* (line 3 in Algorithm 1) is then described. The graphical statistical techniques utilized to analyze a computed multi-dimensional map and reveal interesting energetic features, such as energy basins, are related afterwards. A description of the algorithm employed to build and query the map for energetically-feasible excursions of the target protein sequence *α* between two structures of interest (line 5 in Algorithm 1) concludes this section.



### Extracting variable axes to define a reduced protein structure space

As mentioned in the “Background” Section, a key starting point that has recently allowed EAs to explore complex structures spaces of multi-state proteins is the ability to define variable spaces of reasonable dimensionality to represent protein structure spaces. These variable spaces are extracted based on a statistical characterization of the increasingly rich structural information available in the PDB for a protein sequence *α* and other (variant) sequences similar to it. The characterization is rooted in the principle of conformational selection, summarized next.

*Utilization of structures and the principle of conformational selection:* Let us suppose a structure has been captured for a sequence *β* of a protein in the wet laboratory. This structure represents a thermodynamically-stable state for *β*. If *β* is a variant of a given protein (that is, within a few amino-acid mutations of some neighboring sequence *α*), then the structure that is stable for *β* may possibly be of low-energy in the structure space of some similar sequence *α*. This is in effect the principle of conformational selection [38], under which perturbations such as sequence mutations do not change a protein’s structure space but rather the probabilities (which in turn are related to energies) with which a given sequence is expected to populate the various structural states; in other words, even a structure detected for a variant is expected to be assumed by the WT (and vice-versa) but possibly with a different probability at equilibrium. In summary, known structures of different sequence variants of a protein represent stable and semi-stable structural states of a target sequence.

#### Extracting variable axes via multivariate statistical analysis

Structures in the set $\mathcal {S}_{\text {PDB}}$ are first “converted” into structures of *α* (line 1 in Algorithm 1). The structures are stripped down to CA atoms (effectively discarding all atoms except the central carbon atom – CA atom – of each amino acid in the amino-acid/protein chain). A structure stripped down to the CA atoms is referred to as a CA trace. Since the CA traces corresponding to the set $\mathcal {S}_{\text {PDB}}$ come from sequences possibly different (within a few mutations) from *α*, the amino-acid identities of the CA atoms are replaced with those in the target sequence *α* in each CA trace. The resulting traces are then subjected to a multivariate statistical analysis, PCA, originally described in [27], to extract new variable axes; these are the principle components (PCs) obtained from the PCA (line 2 in Algorithm 1).

In summary, PCA yields new variable axes via an optimal rotation of the original axes that maximizes variance of the data along the new axes [39]s. Ordering of the new axes (PCs) by the variance of the data when projected onto them allows extracting a subset *m* that is typically much less than the original dimensionality of the data, if PCA is indeed effective. Work in [28] shows this to be the case for many multi-state proteins with multi-basin landscapes; with the top two PCs one captures more than 45 % of the variance (which means they can be employed for data visualization) and anywhere between 10−25 PCs allow capturing more than 90 % of the variance. The latter is a reduction by more than ten-fold, as the original structures are of proteins with more than 100 amino acids; stripping them down to their CAs prior to PCA exposes more than 300 Cartesian coordinates on which PCA operates to reveal no more than 25 PCs/coordinates that still capture more than 90 % of the variance.

The variance-ordered PCs are used as variables ({*PC*_1_,…,*PC*_*m*_}) through which to represent a structure. As described in [27], a structure can be represented as an *m*-dimensional point whose coordinates are projections over the *m* axes (obtained via essentially a dot-product operation with each of the axes). The reverse is also possible. Given an *m*-dimensional point, a process that essentially depends on a linear combination of the axes yields 3D coordinates of the CA atoms of the structure corresponding to the point. Going back and forth between the variable space and the structure space of a given protein sequence makes it computationally feasible to map and query the structure space of a protein by techniques that operate on the variable space. Next we describe the EA that explores this variable space to build a PCA-based map of the all-atom energy landscape of a given protein sequence *α*. The map is analyzed and queried for paths by techniques that also operate on the variable space.

### EA building of a multi-dimensional energy landscape map

The EA employed here to map a protein energy landscape is the result of a series of recent works [27–29, 32, 34] that carefully and gradually investigate the impact of various design and implementation decisions regarding the exploration versus exploitation capability of EA-based stochastic search in multi-basin protein energy landscapes. At a conceptual level, the EA evolves a fixed-size population of individuals over generations towards better-fit individuals. Individuals are points in an *m*-dimensional space whose variable axes are the top variance-ordered PCs obtained as described above. The fitness of an individual in the EA is evaluated via the Rosetta *score12* energy function, which measures the all-atom energy of the 3D protein structure corresponding to the individual. The EA is memetic, as an offspring individual obtained by varying a parent individual is subjected to improvement. This is particularly important for individuals that represent molecular structures in order to reduce the number of constraints violated in offspring. An improved offspring is then considered for addition to the map, which is thus dynamically updated during the evolutionary process.

Algorithm 2 summarizes the EA in pseudocode. Rather than specifying a budget in terms of a total number of generations, the algorithm exhausts a total number of fitness or energy evaluations (line 4 in Algorithm 2), as these are the most computationally-demanding step of any algorithm manipulating molecular structures. Once the budget is exhausted, the map is outputted. For completeness, we provide more details of the EA in what follows, paying particular attention to the shaded boxes in Algorithm 2 that constitute the main functional units of the EA. It is worth noting that these units make use of various parameters. In the interest of clarity, these parameters are not listed in Algorithm 2, but we describe them in context and list their values when relating implementation details.



#### Initialization mechanism to seed the EA

Proper initialization is key to exploration. As mentioned above, the CA traces extracted from $\mathcal {S}_{\text {PDB}}$ and “threaded” onto the sequence of interest *α* are the first to be added to the initial population (line 3 in Algorithm 2); the traces are first projected onto the *m* PCs so as to obtain individuals corresponding to them in the variable space. Prior work has considered various strategies to fill in the rest of the population; typically, a higher exploration capability is obtained as the population size increases from 500 to 2,000 individuals (we use 2,000 in this work), and the number of PDB-obtained structures can be significantly smaller than this target population size. In [27, 28], the rest of the population is filled by individuals obtained as offspring of the CA traces via the variation operator (described below). A comprehensive analysis in [29] compares this strategy to two others, one where the rest of the population is filled by individuals drawn at random in the space of the *m* PCs, and another where the initial population does not make use of any of the experimentally-known structures but consists of only individuals drawn at random in the variable space.

Comparison on the average fitness and average diversity (measured via Euclidean distance in the variable/PC space) of a population over generations demonstrates that the strategy where the initial population consists of individuals derived from the experimentally-known traces and individuals drawn at random provides a better balance between exploitation (improvement in average fitness over generations) and exploration (retainment of diversity over generations). In the results described in the “Results” Section, this strategy is employed to seed the EA and obtain the energy landscape maps of various protein sequences.

#### Obtaining offspring via a variation operator

As line 6 in Algorithm 2 indicates, each parent *p* yields an offspring *c*. Variation is introduced in each population through a variation operator (line 7 in Algorithm 2) described in detail in [27, 28]. In summary, a vector is first defined in the PC space; its elements are magnitudes of movement along each of the *m* PCs. The magnitude of the movement along the top PC (that captures the most variance) is sampled uniformly at random in the segment [ −*s*, *s*], where *s* is a user parameter. The magnitudes of the movements along the other PCs respect their variance relative to the variance captured by the first/top PC. While the shape of the space is preserved, the boundaries of the *m*-dimensional embedding of the wet-laboratory traces are not observed, as the ultimate goal is to generate individuals that represent new structures not captured in the wet laboratory for the target sequence *α*.

#### Fixed versus variable budget improvement operator

The obtained offspring *c* is subjected to an improvement operator to obtain a better offspring $\tilde {c}$ (line 8 in Algorithm 2). The process consists of three steps.

First, the offspring, which is a point in the *m*-dimensional PC space, is converted into a set of backbone atoms with coordinates in 3D. This step consists of recovering the CA trace via simple algebra operations (detailed in [28], and then recovering the backbone skeleton from the CA trace via the BBQ backbone reconstruction protocol [40]. The next step subjects the backbone skeleton to the Rosetta *relax* protocol [41]. This protocol is open-source and written in C/C++, which allows easy integration in the EA. The protocol repeatedly guesses coordinates for the side-chain atoms (utilizing the target sequence *α* in the backbone structure fed to it as input) and improves them via a simulated annealing MC search. The result is a 3D structure for all atoms (backbone and side chains) that corresponds to a local minimum in the (Rosetta *score12*) all-atom energy surface of *α*. In the third step, the improved individual $\tilde {c}$ corresponding to the resulting structure is obtained. The CA trace is extracted from the structure, and the trace is projected back onto the space of PCs to obtain $\tilde {c}$. The all-atom Rosetta score (*score12*) is recorded and associated with the $\tilde {c}$. The fact that it is the improved offspring $\tilde {c}$ and not *c* that is added to the set of offspring in line 13 in Algorithm 2 is what makes the EA shown in Algorithm 2 a Lamarckian EA.

In prior work [27–29], a fixed number NrImprovItersMax of iterations of the MC search have been utilized in the improvement operator. Since each iteration exhausts one energy evaluation, the budget of energy evaluations can be effectively wasted by attempting to improve sub-optimal offspring. Recent work in [34] introduces a variable-budget improvement operator, which allocates iterations/energy evaluations based on the promise of an offspring for further improvement. The improvement operator spends only one iteration at a time on improving an offspring *c* until a maximum NrImprovItersMax has been reached on the lineage from a parent to the currently improved offspring. The neighborhood of the currently improved offspring in the Map is analyzed and compared in terms of average fitness to the fitness/energy of the offspring, and a determination is made (via an empirically-determined relationship) on whether the improvement should be terminated prior to reaching the maximum number of iterations. The relationship also determines whether the improved offspring ought to be considered for addition to the Map or not (lines 9–10 in Algorithm 2). If not, the lineage is penalized, as well, so as to remember that this specific region in the variable space ought not to considered further. Lines 11–12 in Algorithm 2) show that the parent of the terminated offspring is replaced with a new individual. While work in [34], generates the new individual at random in the variable space, here we consider an alternative strategy; two parent individuals are selected at random and crossed over (utilizing one-point crossover) to obtain the new individual. These two different strategies are compared in the first set of results related in the “Results” Section.

#### A Sample-based map of a protein energy landscape via a hall of fame

A large population is critical to capture a possibly large set of local minima in a rugged energy landscape. Maintaining all individuals ever generated in memory is not practical; nor is it effective, as many individuals generated during the execution of the EA may be highly structurally-similar. What is needed is a map with a tunable resolution. Work in [29] proposes utilizing the concept of a *hall of fame* to serve as a dynamically-updated, resolution-tunable map of a protein’s energy landscape. The hall of fame is an evolutionary strategy to equip an EA with memory. The algorithm invoked to update it is shown in pseudocode in Algorithm 3.



As Algorithm 3 shows in lines 1–2, if the fitness *f*(*c*) of the individual *c* considered for addition to the map is not below a threshold fitThreshold, *c* is not considered (reflecting the objective to update the map with fit individuals). Otherwise, *c* is considered (line 3) and then compared to neighboring individuals *C* in the map (line 4). If a neighboring individual *C* whose Manhattan distance (in the space of *m* top PCs) falls below the threshold distThreshold but has higher fitness than the fitness of *c*, then the individual is replaced by *c* (lines 5–11). If *c* is similar but does not reside deeper in the local minimum containing *C* (lines 8–9), *c* does not replace *C*. Note that if *c* is not similar to any other individual in the map, it is added, as it represents a new region not currently present in the map. The idea is to update the map with individuals that may represent the same region in the variable space but allow further exploitation of a local minimum and with fit individuals representing novel regions. The distThreshold represents a resolution, as the map is a set of distinct local minima individuals (obtained after improvement) separated by at least the defined threshold distThreshold in the space of PCs. Increasing distThreshold makes the map sparser. Lowering it, provides more detail but also increases the number of individuals in the map.

#### Selection operator

Line 15 in Algorithm 2 invokes the selection operator, where offspring compete with parents for survival. A comparative analysis of various implementations in [28] suggests that a local/decentralized selection operator, where each offspring competes only with parents in a given neighborhood, stalls take-over of the population by most-fit individuals, thus delaying premature convergence. The neighborhood captures the notion of structural similarity, so that offspring only replace structurally-similar parents if they lower Rosetta *score12* energy. Structural similarity is determined efficiently by embedding individuals in an explicit 2D grid over the top two PCs. Cell width is also a user-defined parameter, and values employed here for the construction of the grid are those suggested to be optimal by the comparative analysis in prior work [28]. In recent work [29], a modification is proposed to the local selection operator, which we employ here in applications of the EA analyzed in the “Results” Section. If an offspring does not have any parent individuals in its neighborhood, it survives and is included in the population for the next generation; in prior work [28], such an offspring would compete with all parents.

### Analysis of a multi-dimensional map via graphical statistical techniques

Projections of the multi-dimensional maps onto 2D, while informative (as related in the “Results” Section, may hide interesting energetic features that only appear along the remaining axes. Graphical techniques for visualization of multi-dimensional data are employed here to elucidate interesting energetic features hidden along the different dimensions of the variable space explored by the EA. In all the proteins investigated here, the top 4 PCs capture about 80 % of the dynamics. Therefore, hidden energetic features are sought on at most 4D projections of the computed maps (PC1-PC2-PC3-PC4) by way of two-way conditioned plots.

Two-way conditioned plots provide a way to obtain insight in data patterns related to a 4D domain. Such graphics have a substantial history and are alternatively referred to as multi-window displays, casement displays and co-plots [42–45]. The basic idea is to focus on plots of two variables at a time, conditioning on the other two variables so the basic view is a function of the other variables (or not). Let us refer to the former the primary variables, and the latter as the conditioned-upon variables. Since the PCs are ordered by variance, PC1 and PC2 are used as the primary variables, leaving PC3 and PC4 to be the conditioned-upon variables.

In the two-way conditioned plots we employ to visualize the map along essentially the top 4 PCs, the data is partitioned in 16 subsets that are quartile intervals for PC3 and PC4. Let us consider a specific quartile, Q _*i*_ for PC3 and Q _*j*_ for PC4. The *m*-dimensional individuals in the map are then visualized as follows. All coordinates of an individual along PC5 and on are discarded, and the only individuals retained are those whose third coordinate falls in Q _*i*_ of PC3 and fourth coordinate falls in Q _*j*_ of PC4. This subset resides in a 4D space. In effect, considering the fitness value of each individual adds a fifth dimension. These individuals are visualized in a 2D plot as follows. They are binned in hexagon bins, a popular idea in visualization of multi-dimensional data introduced in [46]. Only the lowest-energy (best) individual is then visualized per bin, plotting it as a 2D point along PC1 and PC2, and color-coding it based on its energy. A blue-to-red color-scheme is employed corresponding to low-to-high energy values.

It is worth noting that the conditioned-plot approach to multi-dimensional data visualization sacrifices much of the resolution of the conditioning (partitioning) variables while retaining much of the resolution for the variables used in the plots. The comparison of juxtaposed plots, however, provides valuable insight into the impact of the conditioning variables. As the results in the “Results” Section relate, a layout of 16 color-coded, hexagon-binned, two-way conditioned plots (16 by combination of each of the quartiles of PC3 with quartiles of PC4) provides an effective way to visualize a 5D view of the maps of energy landscapes constructed by the above-described EA. In particular, the layout allows visualizing how basins elongate along the other dimensions, and where along these dimensions they populate regions not captured in wet laboratories.

### Graph-based query of map for energetically-feasible structural excursions

The map that the EA described above constructs to represent an energy landscape is essentially a (hall of fame) list of (multi-dimensional) individuals with associated fitness values (Rosetta *score12* energies). In order to query the map for ensembles of energetically-feasible structural excursions between any two structures of interest for a protein at hand, the map is first converted into a nearest-neighbor graph. Details are related below. After the map is essentially equipped with connectivity information, any informed graph search algorithm can be employed to query the map for energetically-feasible paths. Below we describe how the nearest-neighbor graph representation of the map is provided with energy-based weights, and how Dijkstra’s shortest-path algorithm [47] is then employed to extract a lowest-cost path connecting two structures of interest from a graph of close to a million vertices. Finally, the rest of the “Methods” Section describes how Dijkstra is employed in an iterative fashion to obtain an ensemble of low-cost paths in order to provide a broader picture of energetically-feasible structural excursions.

#### A nearest-neighbor graph representation of the energy landscape map

The map is converted into a nearest-neighbor graph *G*=(*V*,*E*) as follows. The individuals in the map populate *V*. Each vertex is then connected via edges to *k* other vertices that are its nearest neighbors. Euclidean distance in the *m*-dimensional variable space is used to measure the proximity between two vertices/individuals. The computation of nearest neighbors can be potentially a time-consuming step, but nearest-neighbor search data structures, such as a kd-tree [48], provide a remedy, particularly when the number *N* of data points is much larger than 2^*m*^ (that is, *N*>>2^*m*^), where *m* is the dimensionality of the variable space [49]. We employ a process similar to how the kd-tree organizes data points to support fast nearest-neighbor queries. Specifically, Euclidean distance calculations are terminated earlier than considering all variable axes if the distance already surpasses a dynamic threshold (the latter is updated as neighbors are found).

Since the set *V* can be very large (recall that the distThreshold parameter in the map construction can allow for a highly-detailed map with millions of individuals), the number of nearest neighbors for a vertex is limited to *k*=8. That is, the branching factor for the graph is limited to 8. The graph is directed; a vertex *v* may be among the *k*-nearest neighbors of a vertex *u*, but *u* may not be among the *k* nearest-neighbors of *v*.

While edges are added based on essentially a proximity relationship between vertices, weights or costs associated with them are based on the following energy-/fitness-based relationship: *Cost*(*e*=(*u*,*v*))=*max*{score12(*v*)−score12(*u*),0}. The idea behind this is as follows: If the directed edge *e*=(*u*,*v*) lowers the energy of a protein hopping from *u* to *v*, then this particular *u*→*v* excursion does not require additional energy, as it is a down-hill movement in the landscape. Down-hill movements occur instantaneously per thermodynamics; no build-up of energy is needed to allow the excursion to take place. On the other hand, an up-hill movement, where score12(*v*)>score12(*u*), requires the system to build enough energy in order to cross what is essentially an energy barrier. This way of associating costs with edges is based on the principle of mechanical work, as the cost that would be tallied up with a path of edges would essentially sum only up-hill movements in the landscape; that is, only keep track of the total amount of external work that needs to be performed to give energy to fund such movements. This way of associating costs with edges is shown to assess the relevance of a lowest-cost path as a representative of a structural excursion better than an alternative approach based on the integral cost along the path [50] (and has been used by us before on robotics-inspired protein motion computation [35]).

#### Querying nearest-neighbor graph for low-cost paths

Given two structures *S*_start_ and *S*_goal_, the nearest-neighbor graph can be queried for a path as follows. First, the two structures are projected onto the *m*-dimensional variable space, and their projections *v*_start_ and *v*_goal_ are added to the vertex set. The vertex set is then inspected to find the nearest neighbor *u*_start_ to *v*_start_ and the nearest neighbor *u*_goal_ to *u*_goal_. The directed edges (*v*_start_,*u*_start_) and (*u*_goal_,*v*_goal_) are then added to the set of edges in the graph, with weights are defined above. A path in the enhanced nearest-neighbor graph is then an ordered list of vertices 〈*v*_start_,*u*_start_,…,*u*_goal_,*v*_goal_〉. Dijkstra’s shortest path algorithm is used to compute the lowest-cost path $v_{\texttt {start}} \rightsquigarrow v_{\texttt {goal}}$.

The following modification is carried out in order to produce a physically-realistic lowest-cost path. Since the vertices correspond to individuals obtained via randomized search, the sampling of the structure space is non-uniform; while some structures may have nearest neighbors in very close proximity, this cannot be guaranteed over all structures computed by the EA. Indeed, the most densely-sampled regions will be those in basins due to the nature of EAs. The unintended consequence of non-uniform sampling is that a structure (vertex) may be connected via an edge to a structure (vertex) far away in the structure space. Such connections are valid in the nearest-neighbor graph construction, but they do not provide physically-realistic information regarding structural transitions.

Rather than place additional proximity constraints among a vertex and its *k*-nearest neighbors in the construction of the edge list of the graph, such constraints are imposed when querying the graph for paths; that is, the neighbors of a vertex are a subset of its *k* neighbors in the graph subjected to an additional proximity constraint. A user parameter is considered for this purpose, *max*_*nn*_*dist* (maximum nearest-neighbor distance), and values for this are generated by dividing the Euclidean distance between the individuals corresponding to the start and goal structures by values in the set {15,10,7.5}. The latter can be considered path resolutions, and in the “Results” Section we demonstrate the implication of the resulting different values for *max*_*nn*_*dist*. In summary, a large value allows making large hops in the variable space and associating non-credible costs, such as would be obtained by directly connecting two nearby basins without considering the energetic barrier in between the basins (the equivalent of tunneling through an invisible mountain). A small value is conservative, making much smaller hops and effectively is impacted by the ruggedness of the energy landscape. Very small values of *max*_*nn*_*dist* may result in no paths at all, as no nearest neighbors can be found to meet a very conservative distance criterion.

Dijkstra’s algorithm can be run in an iterative manner to produce more than the lowest-cost path. Once the lowest-cost path is computed, the intermediate vertices (excluding start and goal) in the path are removed from the graph, together with their edges. The remaining graph is queried again for the lowest-cost path, and this process is continued, removing intermediate vertices after identifying a path, until no more paths can be found; that is, the start and goal are now in different connected components. The result of this iterative process is an ensemble of low-cost paths, which are analyzed in the “Results” Section to obtain summary statistics regarding energetically-similar structural excursions of a protein.

### Implementation details

The algorithms for map building and querying are implemented in C/C++, whereas the graphical techniques for analysis of a built map are implemented in R. The EA is run until the budget of 1,000,000 Rosetta *score12* evaluations is exhausted. Population size in the EA is 2,000 individuals. A preliminary analysis in [29] also shows that this population size, combined with the initialization strategy described in above, injects greater diversity in the initial population. The target cumulative variance to obtain *m* PCs is set at 90 %, as in prior work. The step size *s* in the variation operator is set to 1, and NrImprovItersMax in the improvement operator is set to 5. In the map update, fitThreshold is set to 0 Rosetta Energy Units (REUs) for most proteins. For CaM, where Rosetta heavily penalizes non-compact structures, fitThreshold is set to 250 REUs. Also in the map update, distThreshold is set to be twice the minimum Manhattan distance between two wet-laboratory structures of a protein under consideration. In the variable-budget improvement operator, neighbors of an offspring in the map are individuals no more than 1 unit away in Manhattan distance. Prior work on the selection operator indicates that C25 and C49 are reasonable choices that delay premature convergence [28]. Similarly, reasonable values for the grid cell width vary from 1−2 for small proteins less than 100 amino acids and 10 for other longer proteins.

The EA is run on a 16 core red hat Linux box with 3.2 GhZ HT Xeon CPU and 8GB RAM. The cores are employed to parallelize offspring improvements. This results in significant time savings. The experiments reported here are carried out on a 16-core platform, but, since the distribution is embarrassingly parallel, more time savings can be obtained with more cores.

## Results

### Test cases and experimental setup

The proposed methodology is applied to 10 protein sequences, and performance is evaluated both in terms of running time and quality of the maps and structural excursions modeled on each sequence in relation with existing wet- and dry-laboratory evidence on known features of the energy landscapes and equilibrium dynamics.

#### Test cases

The selected test cases are proteins of importance in human biology and with a significant number of structures in the PDB [30]. They are the the superoxide dismutase [Cu-Zn] (SOD1), Calmodulin (CaM), and the WT and disease-related variant forms of the catalytic domain of uncomplexed H-Ras (to which we refer as H-Ras from now on). SOD1 is a 150 amino-acid long protein whose mutations have been linked to familial Amyotrophic lateral sclerosis (ALS) [51]. CaM is an enzyme 148 amino acids long that binds calcium and regulates over 100 target proteins, including kinases, phosphodiesterases, calcium pumps, and motility proteins [52–54]. H-Ras is a 166 amino-acid long protein that mediates signaling pathways that control cell proliferation, growth and development. H-Ras switches between two distinct structural states to regulate its biological activity [55]. Sequence mutations are implicated in various human cancers and other developmental disorders [56], and we study here several single and double mutants (7 variant sequences in all).

#### Data collection and preparation

Due to the implication of these proteins in various critical human diseases, ample structural data of their WT and mutated (variant) sequences exist in the PDB. Only X-ray structures are collected for H-Ras, whereas NMR structures are additionally included for SOD1 and CaM to enrich these datasets. The WT sequence of each of these proteins is obtained from UniProt [57]. Structures obtained from the PDB whose sequence changes by more than 3 amino acid from the WT sequence are discarded. Structures with missing internal amino acids are also discarded. Remaining structures are cropped at the termini, if necessary, so their lengths match the length of the WT. This protocol results in 186 wet-laboratory structures collected for SOD1 from the PDB, 86 for H-Ras, and 697 for CaM. As described in the “Methods” Section, application of PCA to these datasets yields a cumulative variance of 90 % at *m*=25, *m*=10, and *m*=10 PCs for SOD1, CaM, and H-Ras, respectively. A cumulative variance of 45−50 % is captured by the top two PCs on each of these proteins. In the interest of space, the cumulative variance profiles are not shown here, but they have been presented in prior work on analysis of the PC spaces for each of these proteins [27, 28].

#### Experimental setup

**Fig. 1:**
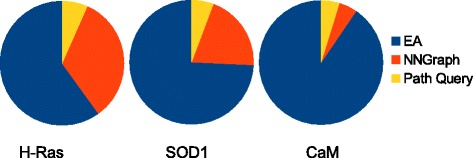
Run time profiling. The break-down of the run time along each of the three main components of the methodology is shown for SOD1, CaM, and H-Ras WT. The path query time refers to the proportion of running time spent on computing the lowest-cost path

The proposed methodology is applied to SOD1 (WT), CaM (WT), and 8 different sequences of H-Ras. The breakdown of the run time of the methodology on each of its components (map building, nearest-neighbor graph computation, and map querying) is shown via pie charts in Fig. 1. The run time of the EA and the size of the maps built on each test case are listed in Table 1. Analysis of the impact of the two different strategies described in the “Methods” Section on how to restart an unpromising lineage is carried out over 3 independent runs of the EA and is related first.

**Table 1:** Map build run time across SOD1, CaM, and H-Ras sequences

Sequence	|Map |	Time (CPU Days)
SOD1	669,102	13
CAM	170,570	9.5
H-Ras WT	890,391	9
H-Ras G12S	699,265	7
H-Ras G12C	704,610	10
H-Ras G12D	694,739	8
H-Ras G12V	649,006	7
H-Ras Q61L	602,893	7
H-Ras C32YS118C	693,567	8
H-Ras R164AQ165V	559,862	7

The analysis then focuses on maps and paths computed on each of the test cases. The analysis on SOD1 and CaM is conducted on color-coded 2D projections of the maps built for each protein and the structural excursions computed for each of them via map queries. This is related next.

The rest of the analysis is on H-Ras, on which there is a wealth of structure data and disease-related mutations. The graphical techniques summarized in the “Methods” Section are applied to the multi-dimensional map generated for H-Ras WT to reveal in detail energetic features that are lost in a 2D projection. Path ensembles, computed as described in the “Methods” Section, are then visualized and analyzed for H-Ras WT and several single- and double-mutant variants. Summary statistics are juxtaposed to supplement the visual comparison of maps and paths. More results are related in the Additional files accompanying this paper. The “Discussion” Section summarizes all results presented on H-Ras to reconcile existing literature and further our understanding of the role of equilibrium structural dynamics on the link between mutations and misfunction in H-Ras variants.

### Exploration versus exploitation: restarting failed lineages with individuals generated at random or via crossover

**Fig. 2:**
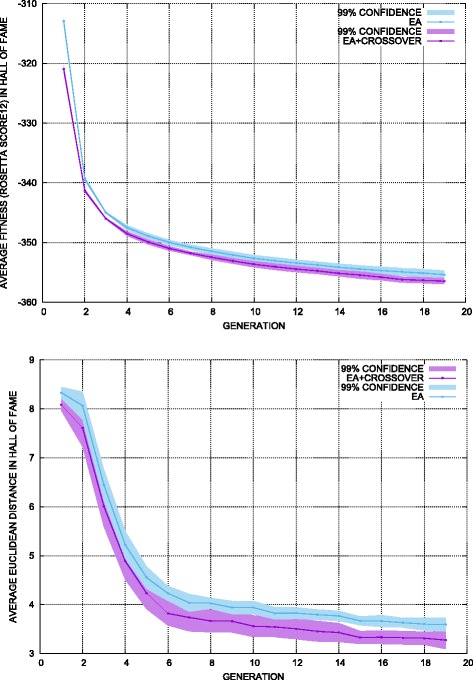
Crossover evaluation. The top panel shows the average fitness/energy among individuals in the hall of fame list (map), as the map is updated over generations. The bottom panel shows the average diversity among individuals in the hall of fame (measured via Euclidean distance) as the map is updated over generations

Two different settings are investigated to restart a failed lineage, generating a new individual at random in the variable space versus generating it via crossover of two parent individuals selected at random in the current population. The EA with each setting is run 3 times, and two measurements are tracked over generations. The first, the average fitness of the growing map (average over fitness values of individuals in the map at a given generation) estimates the exploitation power of the resulting EA. The second, the average diversity among individuals in the growing map (average value over all pairwise Euclidean distances over individuals in the map at a given generation). Figure 2 shows these two measurements for the H-Ras WT map over generations; the 99 % confidence intervals are also shown.

Figure 2 shows that EA with crossover lowers both the average fitness and the average diversity of a growing map faster; that is, the crossover enhances exploitation but lowers exploration. The impact on exploitation is smaller, however, than the impact on exploration. Taken together, this analysis suggests that the EA, where a failed lineage is restarted with an individual generated at random, will be as effective in exploitation and more effective in exploration than when the individual restarting a lineage is generated via crossover. It is worth noting that the differences are not significant; this is expected, as crossover of two individuals that correspond to protein structures is likely to result in similar constraint violations as an individual generated at random in the variable space. The rest of the analysis on the proteins studied here employs the EA where failed lineages are restarted with individuals drawn at random in the variable space.

### Projection-based visualization and analysis of computed energy landscape maps and structural excursions for SOD1 and CaM

One way to visualize computed multi-dimensional maps of energy landscapes is to project individuals in a map onto the top two PCs and color-code the projections based on the Rosetta *score12* energy values; effectively, the 2D projection of a computed map is a 2D projection of the explored *score12* all-atom energy landscape of a protein. Color-coded 2D projections of all individuals ever generated or individuals in a map have been employed by us before to conclude that low-energy regions of an explored protein energy landscape are co-located with projections of experimentally-known structures of a protein [29]; thus, suggesting the ability of a mapping EA operating in a reduced variable space to produce reliable maps of multi-basin energy landscapes. In the following, we show such projections for maps built for SOD1 and CaM. A lowest-cost path is also shown for each protein to demonstrate the ability of the proposed methodology to model structural excursions.

#### Analysis of computed map and basin-basin excursions of SOD1

**Fig. 3:**
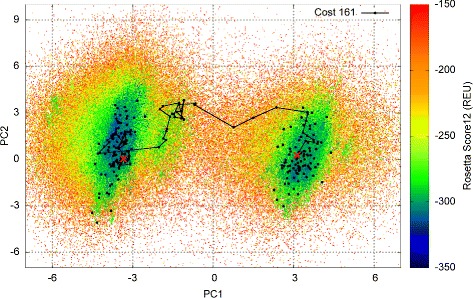
Visualization in 2D of map and a lowest-cost path computed for SOD1. The computed map for SOD1 is projected onto 2D and projections are color-coded by Rosetta *score12* energy values. *Black dots* show projections of experimentally-known structures. A lowest-cost path connecting the two visible basins is additionally drawn. Its cost (in REUs) is also listed

Figure 3 shows the color-coded 2D projection of the map built for SOD1 (WT sequence). The map contains two well-delineated basins. This two-basin feature is related to the phosphorylation event [58], grouping the experimentally-known structures (their PC1-PC2 projections are drawn as black dots) into one of the two basins. The map is queried for a structural excursion between the two basins. Two structures, one residing in each basin, are selected and provided as start and goal to the map querying algorithm in the proposed methodology. The lowest-cost path is computed with a value of *max*_*nn*_*dist* corresponding to about 5.43Å /15 (where 5.43Å is the least-root-mean-squared-deviation – lRMSD – between the two structures, and 15 relates to the sought path resolution (as described in the “Methods” Section. The query is successful; the succession of structures in the path is shown in Fig. 3 by projecting each of the structures onto the top two PCs. The computation of the lowest-cost path points to numerous structures computed by the EA that allow connecting the two basins despite such a conservative (subangstrom) *max*_*nn*_*dist* value. The path also goes nearby various experimentally-known structures in the projection of the energy landscape, which lends more credibility to its validity. Taken altogether, the path demonstrates the ability of SOD1 to undergo structural changes related to the phosphorylation event, effectively switching between two structural states (that separate the experimentally-known structures) during phosphorylation.

#### Analysis of computed map and basin-basin excursions of CaM

**Fig. 4:**
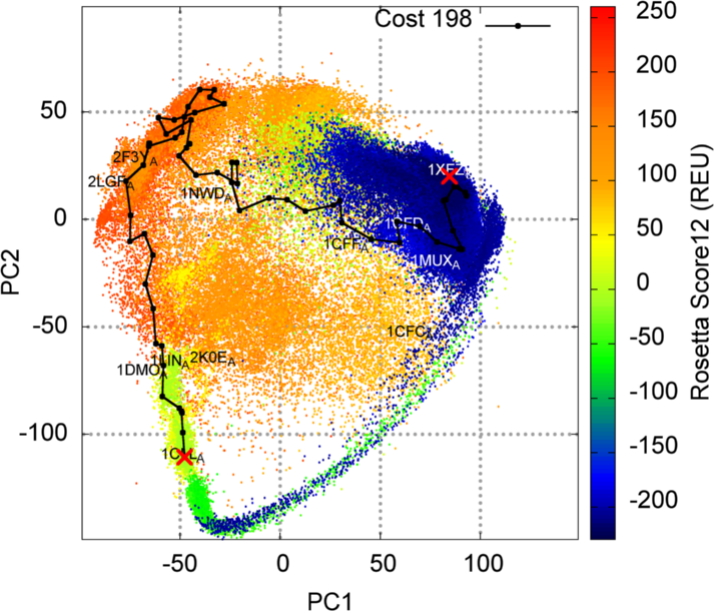
Visualization in 2D of map and a lowest-cost path computed for caM. The computed map for CaM is projected onto 2D and projections are color-coded by Rosetta *score12* energy values. *Black dots* show projections of experimentally-known structures. A lowest-cost path connecting two experimentally-known structures is also drawn; its cost (in REUs) is listed. The PDB ids of the structures the path connects, as well as other experimentally-known structures of interest, are additionally shown where these structures project onto the top two PCs

The ability of the proposed methodology to compute both maps and structural excursions is additionally illustrated on CaM. The color-coded 2D projection of the map is shown in Fig. 4. The map has a characteristic shape, with a hollow region in the middle, indicating the inability of the EA to find low-energy structures in this region. A broad and deep basin is found, populated by many experimentally-known structures, whose PDB ids are annotated. A long narrow strip of low-energy structures is also found. Figure 4 additionally shows the lowest-cost path computed to capture a structural excursion from a compact, closed structure of CaM (PDB id 1XFZ) to the calcium-bound structure (PDB id 1CLL); The lRMSD between the CA atoms of these two structures is 9.5Å, and the shown path is computed with a value of *max*_*nn*_*dist* corresponding to about 9.5Å /10; effectively limiting structural changes between any two successive structures in the path to subangstrom values.

As Fig. 4 shows, the path goes through the calcium-free structure (PDB id 1CFD), passes through compact structures with which CaM binds proteins and peptides (PDB ids 1NWD and 2F3Y) to then reach a structure representative of the calcium-bound state (Ca(2+)-CaM) and in the state bound to myosin light chain kinase (CaM-MLCK) (PDB id 2KOE) just before terminating in the calcium-bound state (PDB id 1CLL). The path confirms that in the succession of structures from the compact state to the calcium-bound state, the domain collapse, re-arrangement, and partial unfolding of the helix linker in CaM are gradual. The succession of structures in the path points to a rearrangement of the domains in the compact state that is needed for CaM to then open up, before populating a semi-open state with a partially-unfolded linker that then further allows it to adopt the open, calcium-bound state. This detailed observation is in agreement with other studies, both those employing MD [59] and others employing robotics-inspired approaches [35].

### Multi-dimensional visualization of computed map of H-Ras WT

Prior work in [29, 34] has analyzed the 2D color-coded projection the H-Ras WT energy landscape in great detail and has concluded that the EA mapping the H-Ras WT energy landscape reproduces the two, large basins corresponding to the two states, On and Off, between which H-Ras switches to regulate its activity in the cell. In addition, the map contains novel low-energy regions not probed in the wet laboratory for H-Ras WT, some of which we analyze in detail here. However, we now do so by considering more than two dimensions.

While important features can be preserved (and thus analyzed and subjected to interpretation) in a 2D projection, other features can be hidden by the projection. Note that, though the top two PCs capture around 45−50 % of the variance of the experimentally-known structures for each protein, essentially 50 % of the dynamics is hidden when projecting the computed maps onto two dimensions. Moreover, the ruggedness of the energy landscape requires careful preparation of the large number of points in a computed map when visualizing them after projection. The above projections for SOD1 and CaM, for instance, are visualized after ordering the points from high to low-energy, so that the low-energy ones are plotted on top of the high-energy ones to prevent occlusion.

**Fig. 5:**
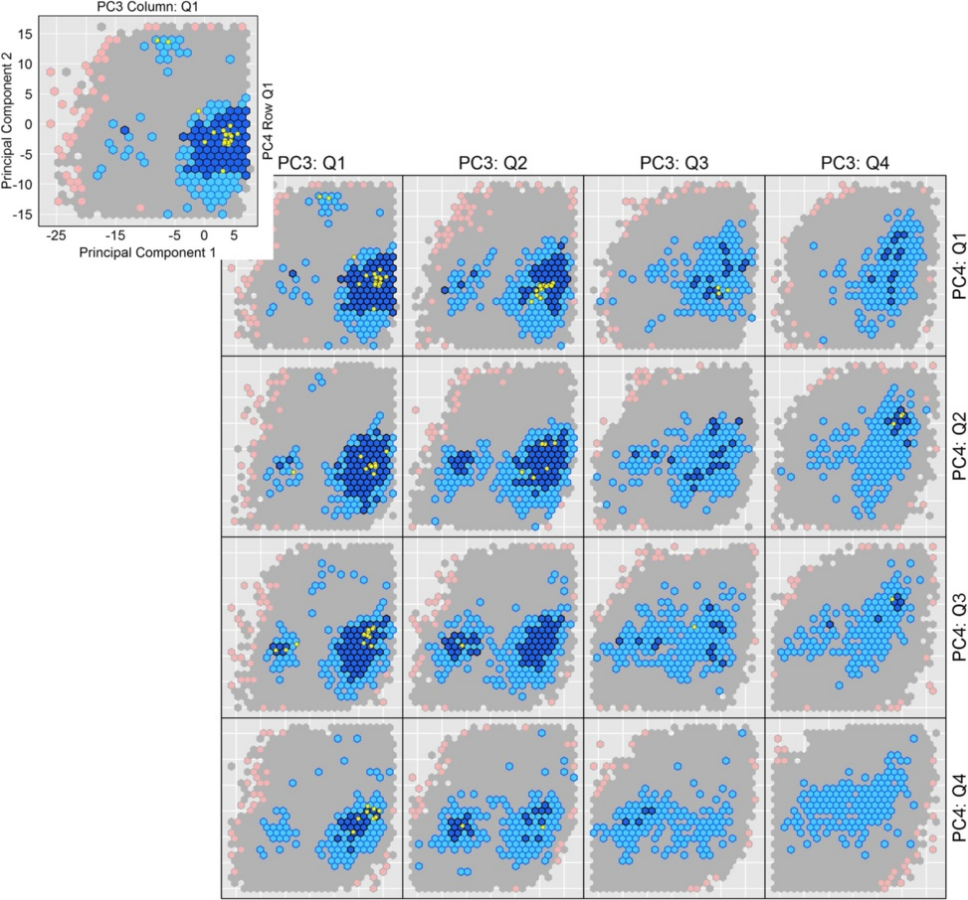
Visualization via conditioning plots of map computed for H-Ras WT. The map computed for the H-Ras WT is visualized via conditioning plots, which plot projections of the hall of fame on PC1 and PC2 conditional on projections on PC3 and PC4. Sixteeen plots are generated, considering combinations of all quartiles of PC3 with all quartiles of PC4. The 4D space in each subplot is discretized via hexagons, plotting for each hexagon only the projection of the lowest-energy individual in the hexagon. The blue-to-red color-coding scheme follows the low to blue energy range. *Dots* in yellow show PC1-PC2 projections of experimentally-known structures of H-Ras WT

Below we relate conditioned plots for the H-Ras WT multi-dimensional map computed by the EA; the plots are constructed as described in the “Methods” Section. The projections are along PC1 and PC2, and the data are conditioned on each of the 4 quartiles of PC3 and PC4. It is worth noting that the top 4 PCs capture more than 75 % of the variance, and thus almost all of the dynamics of H-Ras. The quartile intervals for PC3 and PC4 each have roughly 222,580 data points for H-Ras WT. Table 2 shows the number of cases in common to a chosen quartile of PC3 and a chosen quartile of PC4. The left top panel of Fig. 5 shows a hexagon bin plot along PC1 and PC2 conditioned on the first quartile of PC3 and the first quartile of PC4 (containing 70,908 individuals, as related in Table 2). The color scheme uses color thresholds based on the binned quantiles of cell minimum-energy distributions without subsetting. Quantiles of {0,20,60,99,100} % correspond to Rosetta *score12* values of {−374,−348,−321,−115,−18} REUs. The corresponding color-scheme is {dark blue, light blue, gray, pink }; yellow is reserved to show projections of the experimentally-known structures collected for H-Ras. The right bottom panel of Fig. 5 shows the shift of the minimum-energy patterns, as different subsets of the data are inspected per the 16 two-way conditioned plot layout. In particular, several interesting observations can now be drawn regarding the location of energy basins that the above 2D color-coded projections of the maps did not allow.

**Table 2:** Distribution of H-Ras WT individuals along PC3 and PC4 quartiles

	PC3				
PC4	Q1	Q2	Q3	Q4	
Q1	70,908	57,038	54,913	39,723	222,582
Q2	59,644	57,896	54,655	50,387	222,582
Q3	51,601	56,742	56,261	57,971	222,575
Q4	40,430	50,908	56,748	74,494	222,580
Q5	222,583	222,584	222,577	222,575	890,391

Since the hexagonal binning effectively smooths the ruggedness of the mapped energy landscape, two distinct basins can clearly be seen without the noise due to the ruggedness. The basins are most visible on the PC1-PC2 scatter plots along the second quartile of PC3 and the second or third quartile of PC4 (the [PC3:Q2; PC4:Q2-3] views). The basins reach deep in the energy landscape, as some of the conditioned plots show (for instance, along PC3:Q2 and PC4:Q2-3). The On basin (the dark blue region on the right) persists along all quartiles of PC4 (see first column of the 16-plot layout Fig. 5) but disappears quickly after the second quartile of PC3. No basins are visible on the third and onwards quartiles of PC3 and PC4. The Off basin (dark blue region on the left) is located (and is most visible) on [PC3:Q2; PC4:Q2-3] views.

The experimentally-known structures appear on different quartiles of PC3 and PC4. Specifically, the majority can be found no further than the second quartiles of PC3 and PC4. This observation is particularly interesting, as the portion of the On basin that continues onto the third quartile of PC4 (and second quartile of PC3) does not contain any experimentally-known structures in it. This portion of the On basin is in effect a novel region of the H-Ras WT energy landscape not currently probed in the wet laboratory. As such, the structures in this region constitute a novel stable region that is worth pursuing further in the wet laboratory, particularly in the context of designing drug inhibitors for H-Ras. Similar observations can be drawn regarding portions of the Off basin along specific quartiles, where no experimentally-known structures reside.

### Comparison of maps and basin-basin excursions of H-Ras WT and variants

Maps and structural excursions computed by the proposed methodology on H-Ras are now investigated in greater detail. A comparative setting is pursued to understand dfferences between H-Ras WT and 7 disease-related variants, five of which are single mutants, and two are double mutants. The H-Ras sequences are listed in column 1 in Table 3. The standard naming convention [Code1][Position][Code2] for a single-mutant variant relates that the amino acid named ‘Code1’ (using one-letter amino-acid codes) at position ‘Position’ in the WT is replaced with the amino acid named ‘Code2’ in this particular variant. In other variants, the additional mutations are joined in order of positions; e.g. Y32CC118S.

**Table 3:** Comparison of lowest-cost on →off path across H-Ras WT and variants

Sequence	*m* *a* *x*_*n* *n*_*d* *i* *s* *t* (Å)	Path cost (REU)	Highest Energy (REU)	Nr. Edges
WT	1.45/10	266	–251	90
	1.45/7.5	108	–299	56
G12S	1.45/10	—	—	—
	1.45/ /7.5	130	–230	26
G12C	1.45/10	—	—	—
	1.45/ /7.5	232	–150	66
G12D	1.45/10	—	—	—
	1.45/ /7.5	119	–277	51
G12V	1.45/10	—	—	—
	1.45/ /7.5	109	–276	72
Q61L	1.45/10	—	—	—
	1.45/ /7.5	85	–263	62
Y32CC118S	1.45/10	—	—	—
	1.45/ /7.5	131	–265	54
R164AQ165V	1.45/10	—		—
	1.45/ /7.5	131	–277	72

The EA described in the “Methods” Section is employed to obtain maps for each of the 8 H-Ras sequences. The maps are then queried to compute the lowest-cost paths and other low-cost paths (as described in the “Methods” Section) connecting a structure representative of the On state (PDB id 1QRA) to a structure representative of the Off state (PDB id 4Q21) in each of these 8 H-Ras sequences, effectively modeling the On →Off structural excursion. Two values of *max*_*nn*_*dist* are considered, corresponding to 1.45Å /10 and 1.45Å /7.5, where 1.45Å is the lRMSD between the CA atoms of the structures selected to represent the On and Off states.

**Fig. 6:**
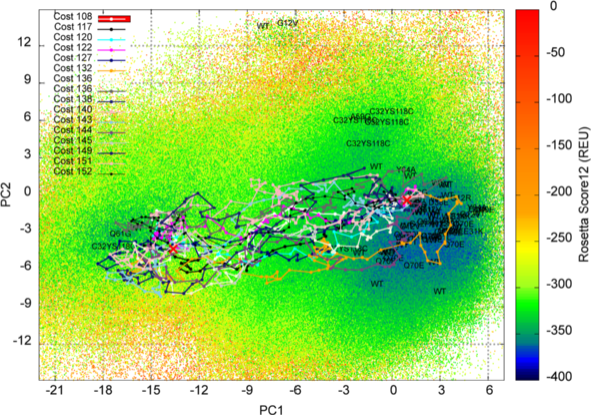
Visualization in 2D of map and paths computed for H-Ras WT. The computed map for H-Ras WT is projected onto 2D and projections are color-coded by Rosetta *score12* energy values. Low-cost paths (costs in REUs are listed) modeling the On →Off structural excursion are also drawn. The projections of experimentally-known structures on the top two PCs are related by showing whether the structures are captured in the wet laboratory for the WT or variants

**Fig. 7:**
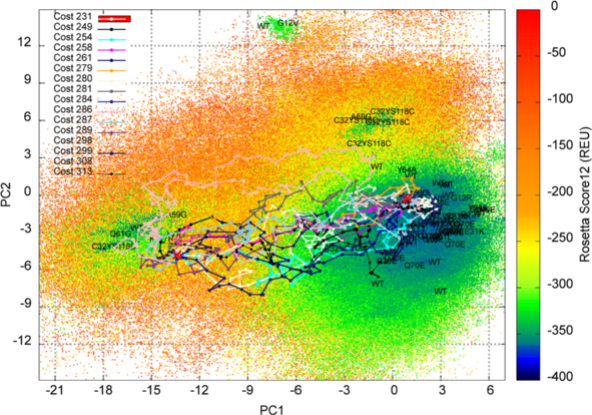
Visualization in 2D of map and paths computed for H-Ras G12C. The computed map for the H-Ras G12C variant is projected onto 2D and projections are color-coded by Rosetta *score12* energy values. Low-cost paths (costs in REUs are listed) modeling the On →Off structural excursion are also drawn. The projections of experimentally-known structures on the top two PCs are related by showing whether the structures are captured in the wet laboratory for the WT or variants

**Fig. 8:**
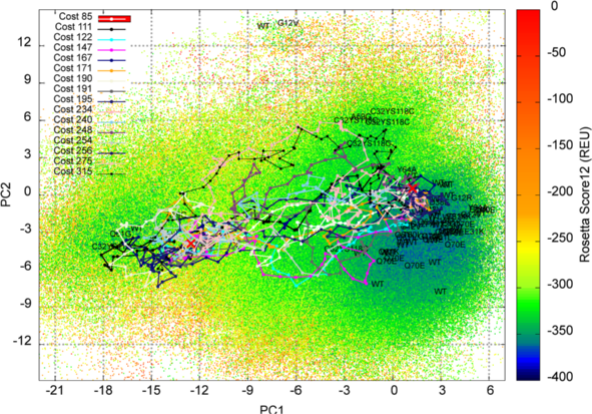
Visualization in 2D of map and paths computed for H-Ras Q61L. The computed map for the H-Ras Q61L variant is projected onto 2D and projections are color-coded by Rosetta *score12* energy values. Low-cost paths (costs in REUs are listed) modeling the On →Off structural excursion are also drawn. The projections of experimentally-known structures on the top two PCs are related by showing whether the structures are captured in the wet laboratory for the WT or variants

**Fig. 9:**
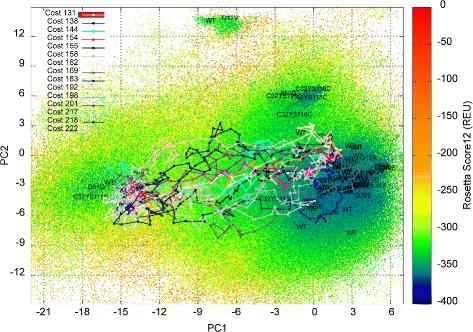
Visualization in 2D of map and paths computed for H-Ras Y32CC118S. The computed map for the H-Ras Y32CC118S variant is projected onto 2D and projections are color-coded by Rosetta *score12* energy values. Low-cost paths (costs in REUs are listed) modeling the On →Off structural excursion are also drawn. The projections of experimentally-known structures on the top two PCs are related by showing whether the structures are captured in the wet laboratory for the WT or variants

Summary statistics for the lowest-cost path and the ensemble of low-cost paths computed on each of the 8 H-Ras sequences are shown in Tables 3 and 4. The low-cost paths (and maps) are also shown on color-coded 2D projections for selected sequences (WT in Fig. 6, G12C in Fig. 7, Q61L in Fig. 8, and Y32CC118S in Fig. 9). Color-coded 2D projections of maps and paths computed for the other variants are related in the Additional files 1, 2, 3 and 4. Summary statistics on paths modeling the (reverse) Off →On structural excursion are related in the Additional files 5 and 6.

**Table 4:** Comparison of ensemble of low-cost on →off paths across H-Ras WT and variants

Sequence	*m* *a* *x*_*n* *n*_*d* *i* *s* *t* (Å)	(*μ*,*σ*)_Cost_ (REU)	(*μ*,*σ*)_Highest Energy_ (REU)	(*μ*,*σ*)_Nr. Edges_
WT	1.45/10	(418.3, 106.)	(–203.5, 25.1)	(101.3, 16.1)
	1.45/7.5	(127.6, 7.90)	(–277.3, 15.2)	(64.2, 15.6)
G12S	1.45/10	—	—	—
	1.45/7.5	(143, 40.9)	(–259, 92)	(73, 22)
G12C	1.45/10	—	—	—
	1.45/7.5	(266.4, 18.2)	(–139.3, 24.4)	(60, 9.8)
G12D	1.45/10	—	—	—
	1.45/7.5	(140.4, 15.5)	(–253.9, 15.9)	(54.6, 8.6)
G12V	1.45/10	—	—	—
	1.45/7.5	(132.3, 13.5)	(–236.3, 83.4)	(64.6, 13.)
Q61L	1.45/10	—	—	—
	1.45/7.5	(161.3, 45.2)	(–240.7, 21.8)	(64.1, 9.2)
Y32CC118S	1.45/10	—	—	—
	1.45/7.5	(158.2, 18.9)	(–257.5, 18.1)	(63.9, 10.2)
R164AQ165V	1.45/10	—	—	—
	1.45/7.5	(159.7, 21.3)	(–245.6, 23.8)	(65.9, 7.1)

Table 3 compares the lowest-cost On →Off path for each of the 8 H-Ras sequences. The cost of the path, the highest energy among structures in the path, and the number of edges in the path are listed in columns 3–5. The lowest-cost path on each H-Ras sequence has been queried off the EA-built map under the two different values for *max*_*nn*_*dist* listed above. The lower value makes it harder to find paths, as indicated by the higher costs and the lack of paths on any sequence but the WT in Table 3. The higher value allows finding more paths, and even lower-cost paths, as the ruggedness of the energy landscape within a ball of radius *max*_*nn*_*dist* is effectively ignored. Since the higher setting of *max*_*nn*_*dist* still corresponds to a very small distance between two successive structures (1.45Å /7.5) and allows obtaining low-cost paths on both WT and variants, the paths shown on 2D projections of the computed maps are those computed for *max*_*nn*_*dist* set to 1.45Å /7.5. Additional file 7 shows the paths that are obtained on H-Ras WT on the lower, more stringent value of 1.45Å /10 for *max*_*nn*_*dist*. The paths are higher in cost, as described above, but they navigate similar regions in the landscape as the paths computed at the less stringent distance of 1.45Å /7.5.

Comparison of the lowest-cost path found for each of the 8 H-Ras sequences at the less stringent distance allows drawing the following conclusion: The majority of the single mutants (with the exception of Q61L and G12V) incur a significantly higher energetic cost for the On →Off structural excursion. This points to a higher energetic barrier separating the On and Off states, which is also visible on many of the 2D projections of the maps built for these variant sequences. The latter is particularly prominent for the G12C variant and can additionally be qualitatively confirmed by comparing the color-coded 2D projection of the H-Ras WT map in Fig. 6 to the 2D projection of the H-Ras G12C map in Fig. 7.

While the results related in Table 3 are informative, they do not take into account the stochasticity of protein motions. Summary statistics on the ensemble of low-cost paths, computed as described in the “Methods” Section, are listed in Table 4 for each of the 8 H-Ras sequences. The comparison of the average cost and average highest-energy along structures in paths generally preserves the ordering of the variants on the lowest-cost paths above. The only variant where this is not the case is Q61L, where a lowest-cost path even lower than in the H-Ras WT can be found, but this path is an outlier compared to the ensemble. The rest of the low-cost paths found for Q61L are much higher in cost, contributing to an average statistic of 161.3 REUs, which is among the highest (the highest average cost is obtained on the G12C variant) when compared to the WT and other variants. This conclusion is in line with qualitative observations made in [33] and similar ones based on visualization of the 2D projection of the energy landscape map in Fig. 8; a high energy barrier between the On and Off basins in the Q61L variant contributes to a structural rigidity in Q61L that effectively causes Q61L to be constitutively activated (always on). The same mechanism is observed on the majority of the variants of H-Ras here.

H-Ras variants where the mutation has a profound impact on the cost of the On →Off structural excursion are those where G12 is mutated to S, C, or D. The higher average path costs over the H-Ras WT for these variants can be also be confirmed by the color-coded 2D projections of the computed maps. For instance, Fig. 7 shows that the entire landscape is elevated in G12C, as many structures become more costly; the On −Off barrier is also higher than in the WT, contributing to the higher average cost for the On →Off excursion. This observation holds on G12S and G12D, as well. In particular, in the G12S variant, whose 2D projection of the map and paths are shown in the Additional file 1, the On basin is very deep, effectively trapping this variant in the On/GTP-binding state. The G12C is also trapped in the On state, but that is due to everything else in the landscape being much more energetically costly. G12V is the only G12* variant where the average cost (and the landscape) is not significantly different from the WT (the paths and the landscape are shown in the Additional file 2). This result is in agreement with an earlier study, where the G12V mutation is proposed to have a subtle effect more on the binding than the energy landscape of the uncomplexed H-Ras variant [33].

Visualization of the maps via color-coded 2D projections reveals an additional interesting energetic feature. G12C, G12S, and the double mutants Y32CC118S and R164AQ165V populate two more regions, distinct from the On and Off basins, with lower-energy structures than the WT, G12V, G12D, and Q61L (the maps for G12S/D and R164AQ165V are provided in the Additional files 1, 3, and 4, respectively). Preliminary evidence of these regions was related by us in prior work on analysis of a first-generation version of our EA on H-Ras WT, G12V, and Q61L [33]. However, in [33], these regions were not exploited as well. These regions, dubbed Conf1 and Conf2 in [33] (Conf1 corresponds to PC1 in [ −3,0] and PC2 in [ 36], and Conf2 corresponds to PC1 in [ −9,−6] and PC2 in [ 1215] in the 2D projections), are populated with very low-energy structures by the EA employed here in the H-Ras G12C, G12S, Y32CC118S, and R164AQ165V variants. The regions constitute new basins, effectively, in these variants. It is interesting that the Conf1 basin emerges only on the G12C/S mutations and not on the G12V mutation, particularly considering that the structure caught in the wet laboratory for the G12V variant projects to this region of the structure space. This is a novel finding of our methodology and suggests that perhaps the relationships regarding shared molecular function profiles between the G12* variants and these double mutants ought to be investigated in greater detail in the wet laboratory.

Finally, it is worth noting that the Conf1 region is populated well by the double mutants, as well. In particular, the Conf1 basin is deeper in the Y32CC118S variant (see Fig. 9), as expected, given that this region contains projections of wet-laboratory structures caught for this variant (thus representing a stable state). This basin is also deep in the R164AQ165V variant (see the Additional file 4). However, both double mutants have a higher energy barrier and a shallower off basin than the WT (see Fig. 9 for the Y32CC118S variant, and the Additional file 4 for the R164AQ165V variant), which results in higher-cost On →Off excursions, as related in Table 4, effectively rigidifying these variants. The latter explains the loss of GTP-binding activity noted for the R164AQ165V variant.

## Discussion

The results presented here suggest that an increasingly detailed picture is emerging of the H-Ras energy landscape. The two-basin feature of the H-Ras energy landscape has been elucidated in both wet and dry laboratories; extensive computational studies by McCammon and colleagues via MD methods have both verified the existence of these two basins and the energy barrier separating them [60]. The two-basin characteristic has also been reproduced via prior versions of the EA algorithm employed here that did not make use of a map but rather analyzed all structures ever generated. The graphical techniques employed in this paper to analyze the map constructed by the proposed methodology provide for the first time a highly detailed view of the multi-dimensional H-Ras energy landscape. In particular, Fig. 5 shows not only how the On and Off basins elongate along the third and fourth dimensions, but also clarify which regions of this multi-dimensional space provide interesting new energetic features not captured in other laboratories. For instance, as described in detail in the “Results” Section, a significant portion of the On basin that continues onto PC4:Q3 (and PC3:Q2) does not contain any experimentally-known structures. Effectively, this represents a new region of the H-Ras energy landscape that is reported to be associated with the stable on structural state by the EA employed here but has yet to be captured in the wet laboratory.

The graphical techniques employed here also allow making comparative observations regarding the depth and width of the On and Off basins. The layout of the 16 two-way conditioned plots in Fig. 5 shows that the On basin is both wider and deeper than the Off basin (this observation can also be made, though less reliably, on the 2D projection of the energy landscape in Fig. 6). The [PC3:Q3-4; PC4:Q3-4] views in Fig. 5 join the two basins, effectively showing the landscape at the higher energy levels. As one proceeds deeper in the landscape, the regions separate to yield the distinct On and Off basins; energy barriers appear along [PC3:Q1-2; PC4-Q*]. The higher width of the On basin points to the higher stability of this basin; that is, the temporal scale of structural excursions of H-Ras from the On to the Off state will be dominated by diffusions within the deep and broad On basin.

The juxtaposition of maps and On →Off structural excursions for the H-Ras WT and the 7 single- and double-mutant variants in the “Results” Section elucidates, among other things, that two new basins emerge on the landscapes of some single- and double mutants, referred to as Conf1 and Conf2. In particular, these are observed to be richly populated in G12C, G12S, and the double mutants, but poorly populated on the other variants and H-Ras WT.

**Fig. 10:**
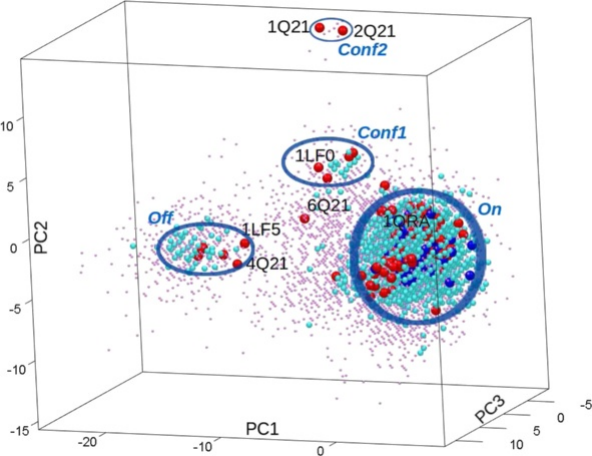
Visualization in 3D of map computed for H-Ras WT. The lowest-energy structures in the map computed for H-Ras WT are shown projected onto the top 3 PCs. Projections of the experimentally-known structures are also drawn, as red spheres of a larger radius. The PDB ids of some of these structures are also shown. The four basins that emerge on the WT and the various variants are also delineated and named per the convention described in the main text

Figure 10 provides a 3D view of the lowest-energy structures (falling in the 1st percentile of the energy distribution) in the map computed for H-Ras WT and the experimentally-known structures by projecting them onto the top three PCs. Picking a lower percentile loses the range of the PCs, which we want to retain in order to show projections of all the experimentally-known structures (drawn as red spheres). The 3D space is partitioned into truncated octahedron cells, as advocated by Carr in [61], and one sphere is drawn at the centroid of each cell. The color and size of a sphere is based on the minimum energy value in the corresponding cell. Three energy intervals are observed for H-Ras WT in this way: [−37.442−367.286] REUs (large blue spheres), (−367.286−350.180] REUs (smaller green-blue spheres), and (−350.180−330.102] REUs (small violet spheres). The interval boundaries correspond to the 0, 0.005, 0.02, and 1 *%* percentiles. The approximate locations of the On, Off, Conf1, and Conf2 basins are delineated in blue in Fig. 10. The PDB ids of selected experimentally-known structures are also annotated.

Comparison of Figs. 5 and 10 shows that the Conf1 and the On basins are merged together by structures with slightly higher energy values (a few REUs in *score12*). In [33], where an early version of the EA is employed (with narrow initialization, no map, and a budget-fixed improvement operator), these structures effectively merging the On and Conf1 basin in the WT are not reported, as the earlier EA has lower exploitation capability. In contrast, the 2D maps of the G12C and the double mutants show Conf1 to be separated by an energy barrier from the On state rather than merged into the On state as in the WT, and to also protrude deeper in the energy landscape than in the WT.

The experimentally-known structure with PDB id 1LF0 sits in the region of the structure space corresponding to the Conf1 basin in the variants and the elongated On basin in the WT (see Fig. 10). This structure has been captured for the H-Ras A59G variant in the active/On state [62]. A 20-ns unbiased MD simulation in [63] has noted that this structure may mediate the On →Off switching in the A59G variant. The intermediate role of this structure is confirmed by the EA here, as this structure is reported to be low-energy for the H-Ras WT and part of the elongated On basin. However, none of the low-cost paths computed for the WT directly employ this structure, as the work-based cost does not promote diffusing in a basin. The in-basin diffusion may explain why this structure has not been captured as an intermediate for the WT during the On →Off excursion in the wet laboratory; it is only in H-Ras variants that an energy barrier gives rise to the distinct Conf1 basin. This barrier may trap variants in Conf1 long enough for this structure to be caught in wet laboratories. Interestingly, another structure, with PDB id 1LF5 (residing in the Off basin in Fig. 10), has been caught for A59G in the Off state.

Taken together, the comparative analysis suggests that the wide On basin retreats in the variants, and an energy barrier splits it into two basins, a narrower On basin and Conf1. The H-Ras WT, once outside the wide On basin, may switch to the stable Off basin or a semi-stable basin observed most clearly in the [PC3:Q1; PC4-Q1] view. This basin sits at the top of the map, in between the On and Off basins, and is referred to as the Conf2 basin. Conf2 is not populated by the lowest-energy structures, but it does contain low-energy structures and two experimentally-known ones. The latter are reported in the PDB under ids 1Q21 and 2Q21. The structure with PDB id 1Q21 is reported as active/On for the WT, whereas that with PDB id 2Q21 is reported as active/On for the G12V variant [64]. The structures are very similar, as noted in [64], and differ mainly in the configuration of the side-chain at position 12, confirming the proximity of these two structures in the PC variable space in Fig. 10 (found at [ −9,−6] in PC1 and [12, 15] in PC2). The work in this paper again confirms that these two structures are functional for the WT from a thermodynamic availability point of view, but perhaps difficult to access within physiological temporal scales due to the high-energy barriers that surround the Conf2 basin. The juxtaposition of the H-Ras WT to the variants in the “Results” Section shows that the Conf2 basin is richly populated in G12C, G12S, and the double mutants. In particular, it is wider and protrudes deeper in the energy landscape for G12C and G12S but not G12V. This is an interesting finding that points to further work in the wet laboratory, as it suggests a novel function regulation mechanism that can be modulated via inhibitors.

The comparison of landscapes and path ensembles across the H-Ras variants provides observations that not only validate and reconcile wet-laboratory findings but may also be useful to further investigation in the wet-laboratory on understanding mutations and designing inhibitors to disrupt aberrant activity [65]. For instance, in addition to the analysis above, a conclusion can be reached regarding the structure with PDB id 6Q21; the asymmetric unit (chain D) of this structure is projected and shown in the 3D view in Fig. 10. This structure is reported for the H-Ras WT in [66]. This unit is in a slightly different structure than the canonical on state (PDB id 1QRA), providing in [66] the earliest evidence of the structural flexibility of H-Ras WT. Figure 10 shows that the structure captured for the WT in PDB id 6Q21 is in a region of the energy landscape populated by low-energy structures part of the elongated On basin in H-Ras WT. The increases in costs reported here associated with structural excursions of H-Ras variants correspond to increases in the time it takes to undergo the excursion at equilibrium. Since molecular recognition events occur at carefully-calibrated temporal scales, any disruption to temporal scales is consequential for molecular recognition events, and thus normal biological activity in the cell.

## Conclusions

This paper introduces a novel methodology to map a protein’s energy landscape and model equilibrium dynamics. Rather than simulate the dynamics of the covalently-bound network of atoms in a protein molecule, the proposed methodology relies on stochastic search to obtain a sample-based representation of the constrained structure space relevant for the dynamics, and then employs discrete search structures to summarize the dynamics. An EA is employed to map the multi-dimensional energy landscape of a protein, and a nearest-neighbor graph representation of the map is then queried to reveal energetically-feasible successions of structures mediating structural excursions of interest. Analysis of applications on several proteins of importance to human biology and disease suggests the proposed methodology is useful in understanding the relationship between protein structure, dynamics, and function with a practical computational budget.

While obtaining a detailed characterization of protein equilibrium dynamics remains a challenge in silico, the work here exploits the wealth of structure data and novel randomized search strategies to enhance exploration of the thermodynamically-available structure space. The exploitation of structure data is a powerful and timely mechanism to map the structure space of a protein. The availability of wet-laboratory structures representing semi-stable and stable structural states for many proteins allows formulating algorithms that can map energy landscapes within a reasonable computational budget, as demonstrated here.

The work presented here opens up several promising directions for future research. One direction concerns lowering the dependency of the methodology on sufficient structure data, as well as expanding its applicability to systems where experimentally-known structure reside in a non-linear low-dimensional space. The first can be addressed via techniques such as Normal Mode Analysis, already integrated with some success in robotics-inspired modeling of protein motions [67–70]. The second can be addressed via linear dimensionality reduction techniques.

Another direction of future research concerns improving the predictions of the locations and depths of mapped basins by employing various energy functions. This direction aims to increase the reliability of in-silico predictions. Considering multiple energy functions remains challenging, however, as considerable recoding efforts are required to efficiently integrate such functions in in-house code.

All data obtained by the proposed methodology and analyzed here are available to the research community upon request. Similarly, any components of the proposed methodology can be shared as linux binaries.

## Additional files

Additional file 1 Visualization in 2D of Map and Paths Computed for H-Ras G12S. The computed map for the H-Ras G12S variant is projected onto 2D and projections are color-coded by Rosetta *score12* energy values. Low-cost paths (costs in REUs are listed) modeling the On →Off structural excursion are also drawn. The projections of experimentally-known structures on the top two PCs are related by showing whether the structures are captured in the wet laboratory for the WT or variants. (PDF 1597 kb)

Additional file 2 Visualization in 2D of map and paths computed for H-Ras G12V. The computed map for the H-Ras G12V variant is projected onto 2D and projections are color-coded by Rosetta *score12* energy values. Low-cost paths (costs in REUs are listed) modeling the On →Off structural excursion are also drawn. The projections of experimentally-known structures on the top two PCs are related by showing whether the structures are captured in the wet laboratory for the WT or variants. (PDF 1772 kb)

Additional file 3 Visualization in 2D of map and paths computed for H-Ras G12D. The computed map for the H-Ras G12D variant is projected onto 2D and projections are color-coded by Rosetta *score12* energy values. Low-cost paths (costs in REUs are listed) modeling the On →Off structural excursion are also drawn. The projections of experimentally-known structures on the top two PCs are related by showing whether the structures are captured in the wet laboratory for the WT or variants. (PDF 1823 kb)

Additional file 4 Visualization in 2D of map and paths computed for H-Ras R164AQ165V. The computed map for the H-Ras R164AQ165V variant is projected onto 2D and projections are color-coded by Rosetta *score12* energy values. Low-cost paths (costs in REUs are listed) modeling the On →Off structural excursion are also drawn. The projections of experimentally-known structures on the top two PCs are related by showing whether the structures are captured in the wet laboratory for the WT or variants. (PDF 1648 kb)

Additional file 5 Comparison of lowest-cost off →on path across H-Ras WT and variants. Column 1 lists the different H-Ras sequences investigated. The two different values used in the query of the map are listed in column 2. The cost of the path, the highest energy among structures in the path, and the number of edges in the path are listed in columns 3–5. (PDF 13 kb)

Additional file 6 Comparison of ensemble of low-cost off →on paths across H-Ras WT and variants. Column 1 lists the different H-Ras sequences investigated. The two different values used in the query of the map are listed in column 2. Columns 3–5 show summary statistics, such as mean and standard deviation, are reported for path cost, highest energy over structures in a path, and the number of edges in a path. (PDF 17 kb)

Additional file 7 Visualization in 2D of map and (finer-resolution) paths computed for H-Ras WT. The computed map for H-Ras WT is projected onto 2D and projections are color-coded by Rosetta *score12* energy values. Low-cost paths (costs in REUs are listed) modeling the On →Off structural excursion are also drawn, now using a more stringent distance criterion for two successive structures in the path. The projections of experimentally-known structures on the top two PCs are related by showing whether the structures are captured in the wet laboratory for the WT or variants. (PDF 2109 kb)
