# Supplementary figures and images for: Computing energy landscape maps and structural excursions of proteins
Source: BMC Genomics. 2016 Aug 18;17(Suppl 4):546. doi: 10.1186/s12864-016-2798-8 (PMC5001232; doi:10.1186/s12864-016-2798-8)

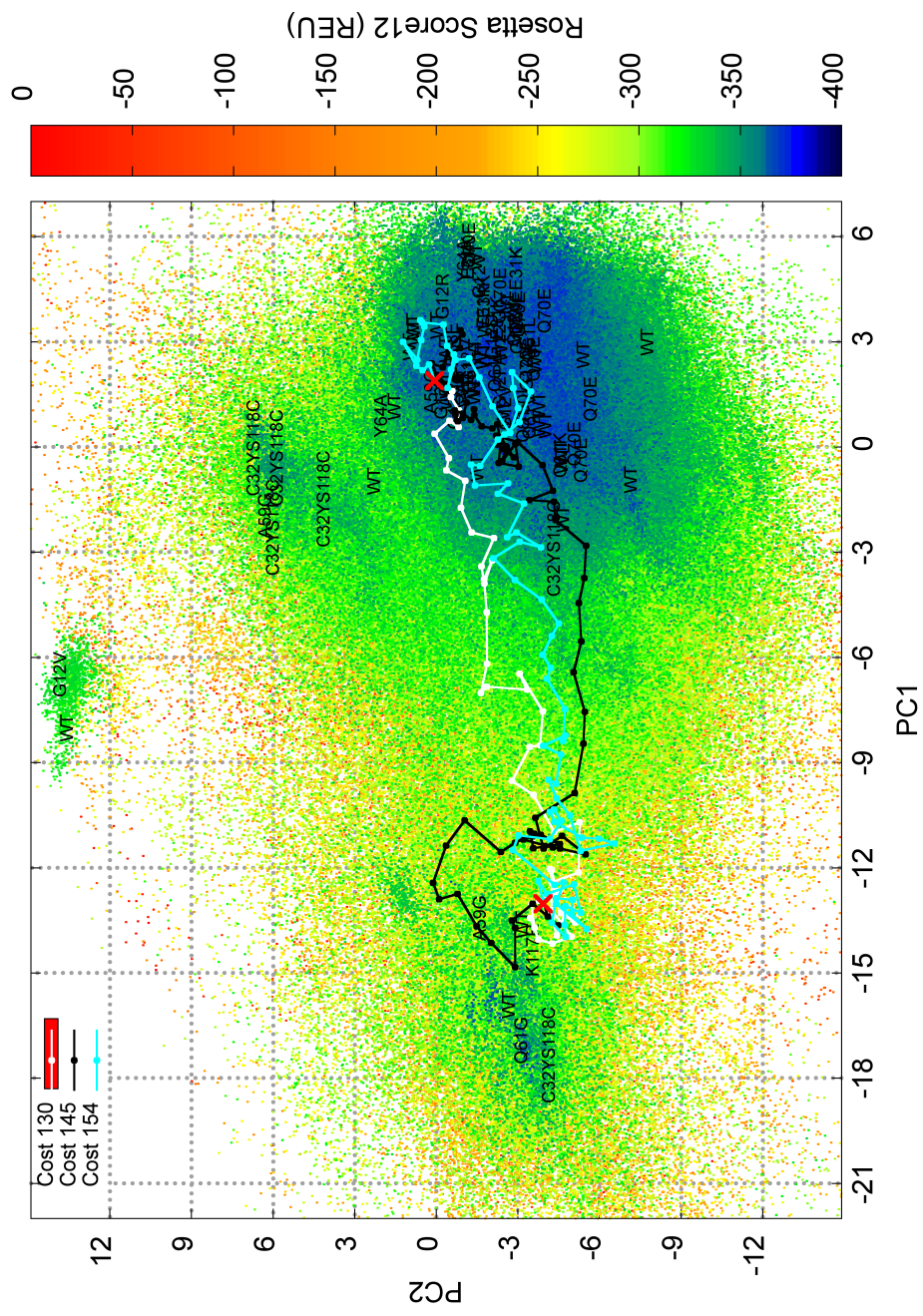

Supplement: Additional file 1 — Visualization in 2D of Map and Paths Computed for H-Ras G12S. The computed map for the H-Ras G12S variant is projected onto 2D and projections are color-coded by Rosetta score12 energy values. Low-cost paths (costs in REUs are listed) modeling the On →Off structural excursion are also drawn. The projections of experimentally-known structures on the top two PCs are related by showing whether the structures are captured in the wet laboratory for the WT or variants. (PDF 1597 kb) [file 12864_2016_2798_MOESM1_ESM.pdf]

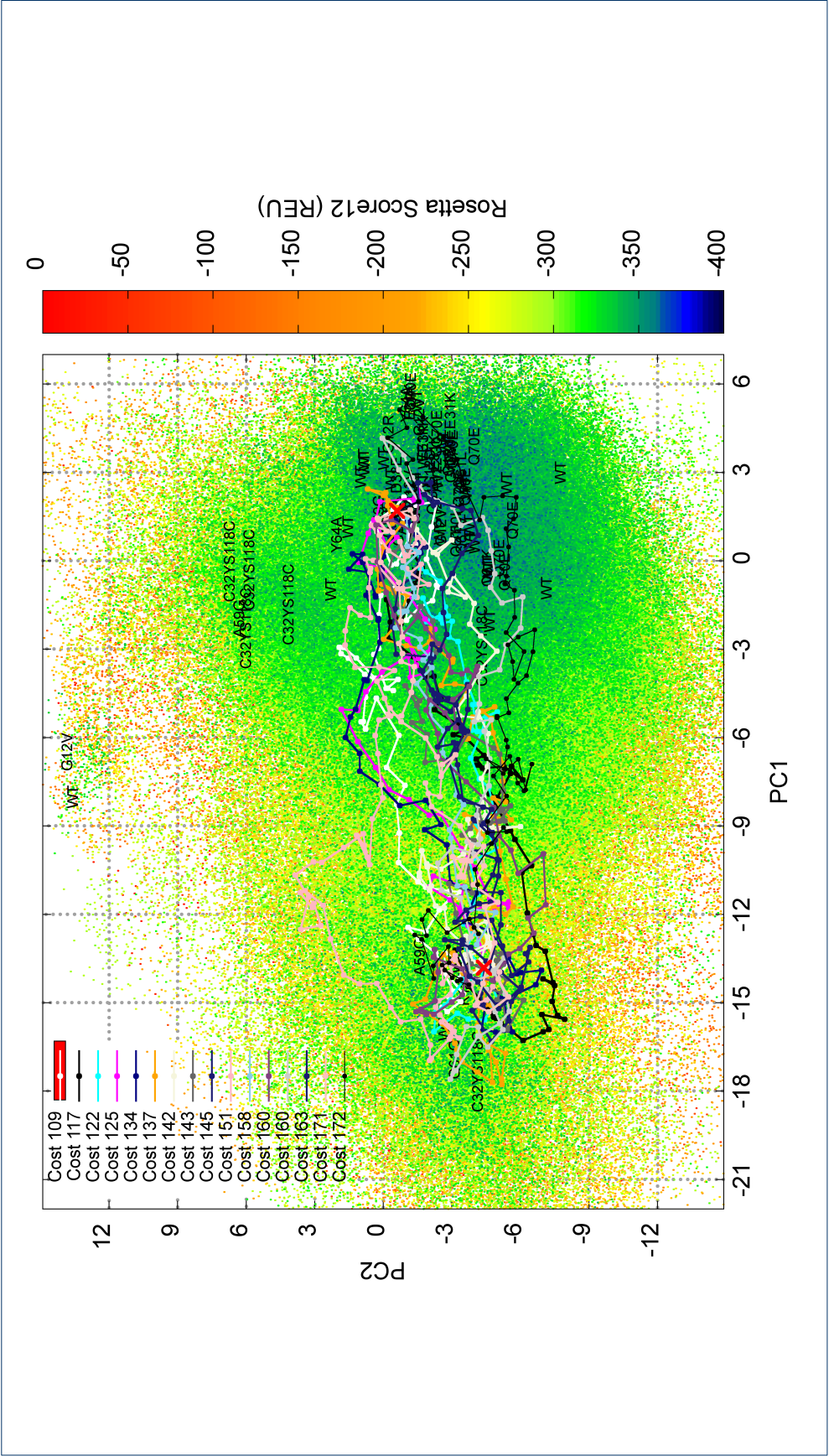

Supplement: Additional file 2 — Visualization in 2D of map and paths computed for H-Ras G12V. The computed map for the H-Ras G12V variant is projected onto 2D and projections are color-coded by Rosetta score12 energy values. Low-cost paths (costs in REUs are listed) modeling the On →Off structural excursion are also drawn. The projections of experimentally-known structures on the top two PCs are related by showing whether the structures are captured in the wet laboratory for the WT or variants. (PDF 1772 kb) [file 12864_2016_2798_MOESM2_ESM.pdf]

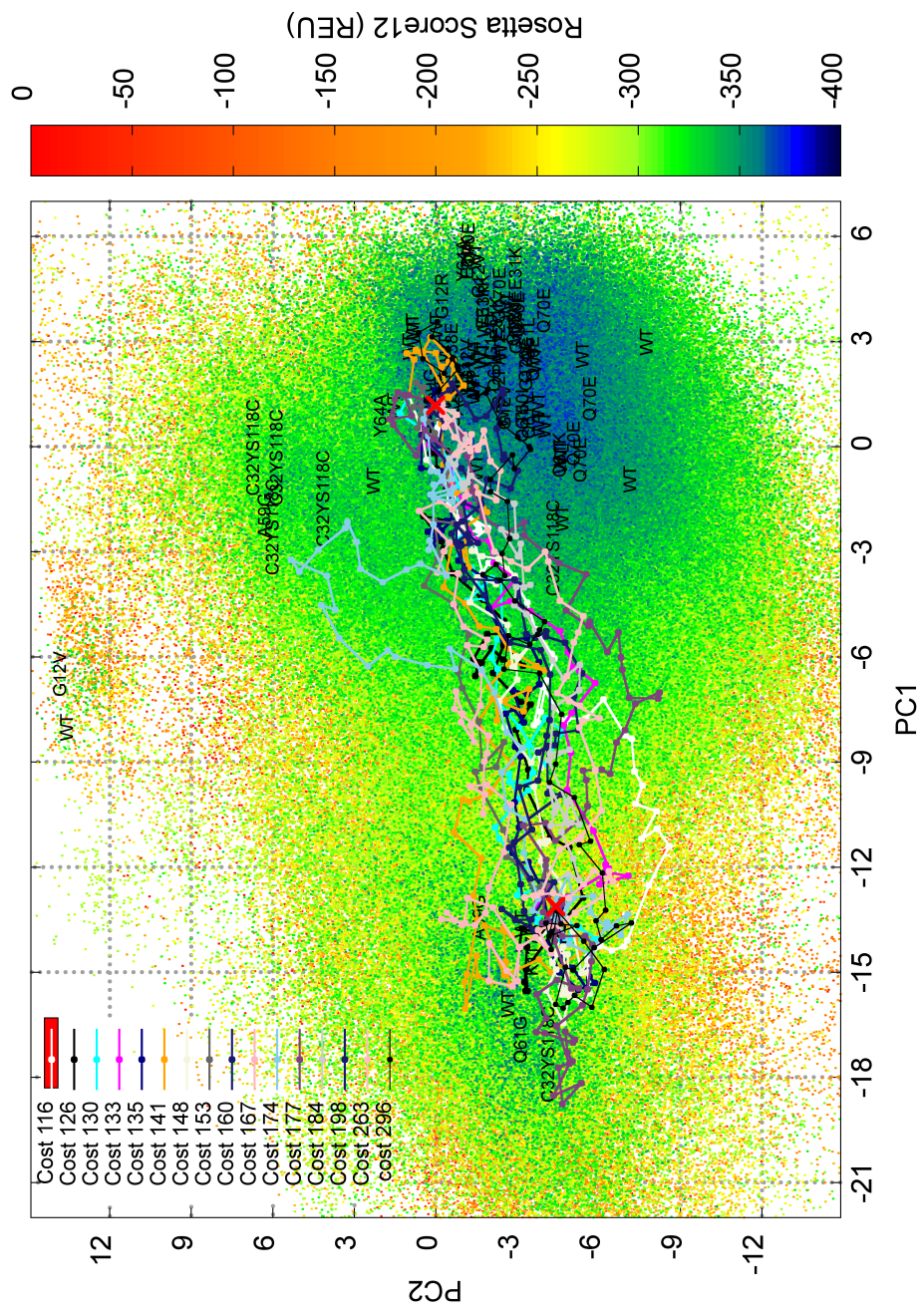

Supplement: Additional file 3 — Visualization in 2D of map and paths computed for H-Ras G12D. The computed map for the H-Ras G12D variant is projected onto 2D and projections are color-coded by Rosetta score12 energy values. Low-cost paths (costs in REUs are listed) modeling the On →Off structural excursion are also drawn. The projections of experimentally-known structures on the top two PCs are related by showing whether the structures are captured in the wet laboratory for the WT or variants. (PDF 1823 kb) [file 12864_2016_2798_MOESM3_ESM.pdf]

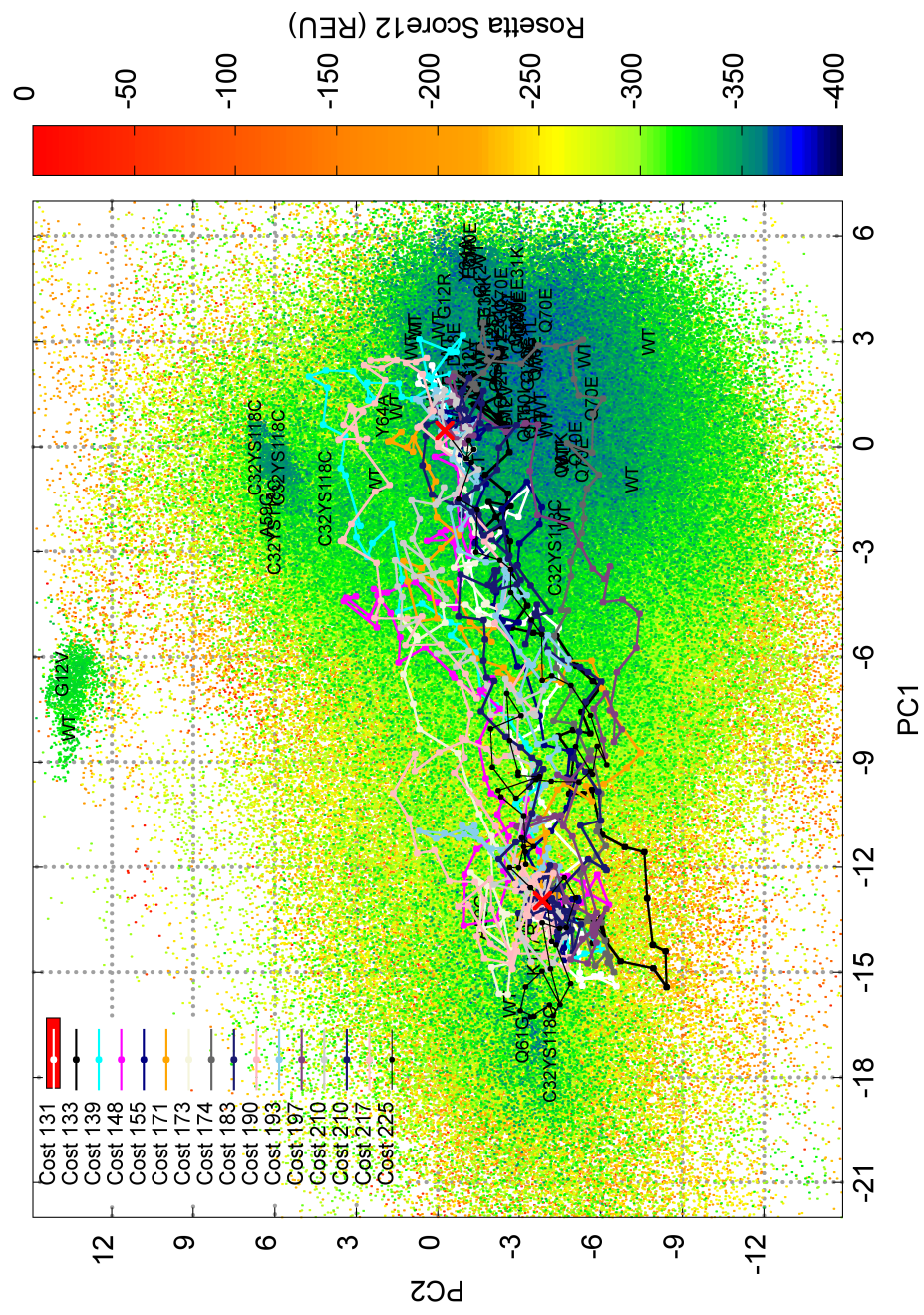

Supplement: Additional file 4 — Visualization in 2D of map and paths computed for H-Ras R164AQ165V. The computed map for the H-Ras R164AQ165V variant is projected onto 2D and projections are color-coded by Rosetta score12 energy values. Low-cost paths (costs in REUs are listed) modeling the On →Off structural excursion are also drawn. The projections of experimentally-known structures on the top two PCs are related by showing whether the structures are captured in the wet laboratory for the WT or variants. (PDF 1648 kb) [file 12864_2016_2798_MOESM4_ESM.pdf]

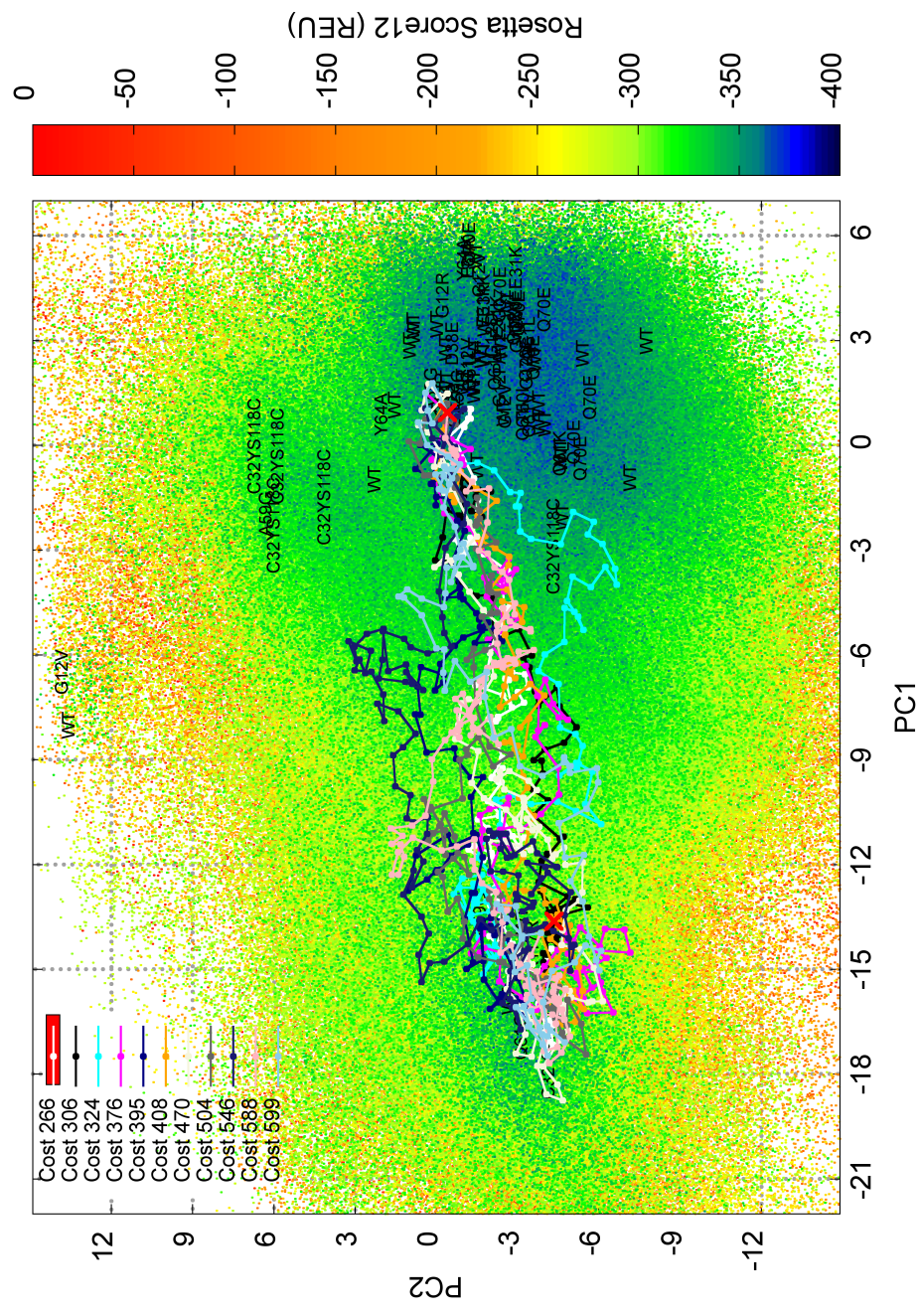

Supplement: Additional file 7 — Visualization in 2D of map and (finer-resolution) paths computed for H-Ras WT. The computed map for H-Ras WT is projected onto 2D and projections are color-coded by Rosetta score12 energy values. Low-cost paths (costs in REUs are listed) modeling the On →Off structural excursion are also drawn, now using a more stringent distance criterion for two successive structures in the path. The projections of experimentally-known structures on the top two PCs are related by showing whether the structures are captured in the wet laboratory for the WT or variants. (PDF 2109 kb) [file 12864_2016_2798_MOESM7_ESM.pdf]
