# Supplementary material for: Computing energy landscape maps and structural excursions of proteins
Source: BMC Genomics. 2016 Aug 18;17(Suppl 4):546. doi: 10.1186/s12864-016-2798-8 (PMC5001232; doi:10.1186/s12864-016-2798-8)
Supplement: Additional file 5 — Comparison of lowest-cost off →on path across H-Ras WT and variants. Column 1 lists the different H-Ras sequences investigated. The two different values used in the query of the map are listed in column 2. The cost of the path, the highest energy among structures in the path, and the number of edges in the path are listed in columns 3–5. (PDF 13 kb) [file 12864_2016_2798_MOESM5_ESM.pdf]

Sequence	max_nn_dist (Å)	Path Cost (REU)	Highest Energy (REU)	Nr. Edges
WT	1.45/10	215	-251	94
	1.45/7.5	71	-299	56
G12S	1.45/10	–	–	–
	1.45/7.5	72	-271	71
G12C	1.45/10	–	–	–
	1.45/7.5	63	-115	51
G12D	1.45/10	–	–	–
	1.45/7.5	47	-267	59
G12V	1.45/10	–	–	–
	1.45/7.5	77	-277	69
Q61L	1.45/10	–	–	–
	1.45/7.5	123	-261	49
Y32CC118S	1.45/10	–	–	–
	1.45/7.5	58	-269	70
R164AQ165V	1.45/10	–		–
	1.45/7.5	58	-269	69
