# Supplementary material for: Computing energy landscape maps and structural excursions of proteins
Source: BMC Genomics. 2016 Aug 18;17(Suppl 4):546. doi: 10.1186/s12864-016-2798-8 (PMC5001232; doi:10.1186/s12864-016-2798-8)
Supplement: Additional file 6 — Comparison of ensemble of low-cost off →on paths across H-Ras WT and variants. Column 1 lists the different H-Ras sequences investigated. The two different values used in the query of the map are listed in column 2. Columns 3–5 show summary statistics, such as mean and standard deviation, are reported for path cost, highest energy over structures in a path, and the number of edges in a path. (PDF 17 kb) [file 12864_2016_2798_MOESM6_ESM.pdf]

Sequence	max_nn_dist (Å)	$(\mu, \sigma)_{\mathrm{Cost}}$ (REU)	$(\mu, \sigma)_{\mathrm{HighestEnergy}}$ (REU)	$(\mu, \sigma)_{\mathrm{Nr.Edges}}$
WT	1.45/10	(354.9, 94.)	(-182.2, 69.)	(92.2, 18.1)
	1.45/7.5	(82.6, 5.7)	(-257.9, 35.2)	(70.3, 23.4)
G12S	1.45/10	–	–	–
	1.45/7.5	(130.9, 42.3)	(-254.9, 24.2)	(86.9, 17.7)
G12C	1.45/10	–	–	–
	1.45/7.5	(79.7, 10.5)	(-116.4, 14.2)	(60.2, 11.1)
G12D	1.45/10	–	–	–
	1.45/7.5	(60, 18.4)	(-258.9, 12.0)	(66.5, 4.9)
G12V	1.45/10	–	–	–
	1.45/7.5	(102.2, 15.1)	(-258.9, 10.8)	(82.2, 18.6)
Q61L	1.45/10	–	–	–
	1.45/7.5	(195.2, 29.8)	(-250.6, 13.6)	(82.2, 18.6)
Y32CC118S	1.45/10	–	–	–
	1.45/7.5	(97.5 27.5)	(-261.3, 19.1)	(70.2, 13.0)
R164AQ165V	1.45/10	–	–	–
	1.45/7.5	(77, 16.8)	(-250.5, 16.4)	(65.3, 2.1)
